# Qualifying and quantifying analysis of biodegradable biocomposite pots to unravel structural insights for sustainable agricultural applications

**DOI:** 10.1038/s41598-026-60732-2

**Published:** 2026-07-07

**Authors:** Manar E. Elashry, Elsayed G. Khater, Samir A. Ali

**Affiliations:** Agricultural and Biosystems Engineering Department, Faculty of Agriculture, Benha University, Benha, Egypt

**Keywords:** Fiber composites, Natural fillers, Sugarcane bagasse, Mercerization, Biocomposite materials, Biodegradable cultivating pots, Palm wax, Morphological properties, Chemical properties, Surface topography, Plant growth, Sustainable agriculture, Biotechnology, Engineering, Environmental sciences, Materials science, Plant sciences

## Abstract

This study investigates the chemical composition, structural evolution, and agronomic performance of biodegradable cultivating pots fabricated from sugarcane bagasse reinforced with natural lignocellulosic fillers. Alkaline pretreatment using sodium hydroxide enhanced fiber–matrix interfacial bonding through lignin and hemicellulose reduction, as confirmed by FTIR and XRD analyses. SEM–EDX characterization revealed high carbon content (34.5–54.4%) and the presence of essential plant nutrients, indicating favorable structural biodegradability and nutrient-recycling potential. Greenhouse trials demonstrated a 13% increase in pepper yield for plants cultivated in treated biocomposite pots, thus proving their agronomic performance efficacy. In addition, quantitative image-based analysis of SEM micrographs using ImageJ and OriginPro enabled the assessment of microstructural homogeneity, grain intensity, surface porosity, and surface topography, including roughness and waviness, supported by 2-D fast Fourier transformation. This single-step analytical framework establishes a transferable pathway for evaluating alternative fillers and binders and enables the rational design of customized biodegradable cultivating pots tailored to specific plant requirements, thereby supporting circular economy strategies and future sustainable agricultural systems.

## Introduction

The global agricultural sector faces dual challenges: reducing its environmental footprint and sustaining food production for a growing population. Biodegradable cultivating pots address these challenges by replacing conventional plastics with renewable materials that decompose into nutrient-rich compounds. In recent years, there has been an increasing exploration and utilization of renewable plant resources, primarily driven by the diminishing availability of non-renewable resources^1^.By integrating natural fillers such as sugarcane bagasse and peat moss with eco-friendly binders, these pots promote responsible resource utilization and minimize waste. Additionally, the reduction in greenhouse gas emissions associated with their production and biodegradation supports global climate action efforts.

The effectiveness of agro-waste pretreatment depends on maintaining the native structure of biomass components while delignifying the biomass. This ensures energy efficiency, cost-effective operation, and reduction in biomass particle size^2^. Pretreatment methods fall into three broad categories: physical, chemical, and biological^3^. Physical pretreatment involves reducing biomass particle size using milling, extruder screws, grinders, and ultraviolet or microwave radiations^4^. Chemical pretreatment disrupts biomass structure by breaking intra- and interpolymer bonds within primary organic components^5^, employing compounds such as acids, organic solvents, alkalis, and ionic liquids. Biological pretreatment uses enzyme-producing fungi to degrade cellulose, hemicellulose, and lignin content in biomass^6^.

Due to the high moisture sensitivity of natural fibers, moisture absorption can lead to delamination between the matrix and fiber, significantly reducing composite mechanical properties. This is attributed to the polar and hydrophilic nature of natural fibers, creating conditions favorable for water absorption. Environmental factors such as sun exposure, rainfall, soil conditions, and water availability during plant growth, as well as processing and production conditions, further influence natural fiber performance. The variability in these conditions may result in differing properties and performance of natural fibers, even within the same cultivation population or harvesting season. Another limitation is the low thermal stability of natural fibers. However, physical and chemical modifications offer viable solutions to address these challenges in the utilization of natural fibers in composites^7^.

As summarized in Table 1, a diverse range of natural fillers and binding agents were employed in the fabrication of biodegradable cultivating pots, necessitating a systematic approach to qualify and quantify their structural and functional properties. Such evaluation is essential for establishing reliable classification criteria and enabling the rational adaptation of biocomposite formulations to meet the requirements of plants at different growth stages. Chemical modification through sodium hydroxide pretreatment plays a critical role in improving filler–matrix compatibility by reducing lignin and hemicellulose content, thereby enhancing structural cohesion and moderating hydrophilicity, as elucidated through FTIR and XRD analyses. Accordingly, this study examines the chemical characteristics, functional group evolution, and morphological features of biodegradable cultivating pots, providing a quantitative framework that links material structure to agronomic functionality and supports the development of sustainable alternatives to conventional plastic containers.

**Table 1:** Materials used in biodegradable pots.

Biomass raw material	Binding matrix	Measuring parameters	Outcomes remarks	Reference
Sugarcane bagasse, compost, peatmoss, activated carbon, & vermiculite	Palm wax Lanette wax	*Physical properties:* water absorption, density & porosity *Mechanical properties:* tensile stress, tensile modulus & elongation at break *Optimization of the properties:* TOPSIS-Taguchi *Biodegradation properties:* disintegration, mineralization & kinetic modelling *Cultivation:* growth and yield parameters	Among all formulations, the palm wax–based pot demonstrated superior performance, with a tensile strength of 14 MPa and 66% disintegration after 90 days of composting	^8–10^
Waste newspapers	Sweet potato (*shochu*) distillation lees & chemical fertilizers	*Physical properties:* weight, thickness, and density *Mechanical properties:* compressive and tensile strength *Morphological properties:* scanning electron microscopy (SEM) *Chemical & biological properties:* Koshino’s Fertilizer Analysis Method for (nitrogen) N, (phosphorous) P, (potassium) K, calcium (Ca) and magnesium (Mg) *Cultivation:* dry weights of the root, stem, leaf, and fruit	Pots provide fertilizer without generating waste	^11^
Peat, grape residue & cellulosic fibers	Wet strength resin (Kymene 611) & nutritive additives: (*urea and dibasic ammonium phosphate* for N & P release; *barax and ammonium molybdate* for microelements release as boron and molybdenum; *zinc sulphate and copper sulfate* for zinc & copper release; *potassium nitrate* for K release)	*Mechanical properties:* puncture strength *Biodegradation:* weight loss percentage of pots & CO_2_ respiration rate *Cultivation:* rhizosphere intensity assessment of tomato and lettuce seedlings	The biodegradation rate reached 45% within 141 days of decomposition Planting into tomato seedlings resulted in a higher rate of biodegradation compared to lettuce seedlings, likely due to the more intense and robust rhizosphere activity associated with tomatoes	^12^
Tomato & hemp fibers	Sodium alginate & polyglycerol plasticizer	*Physical properties:* density, porosity, water uptake & permeability *Mechanical properties:* flexural & puncture test *Morphological properties:* scanning electron microscopy (SEM) *Biodegradation:* CO_2_ production *Cultivation:* plant root & shoot height	The pots broke down completely in 16 days The root radially perforated the wall of pots with no observed rot	^13^
ـــ	Thermoplastic starch produced from native cassava starch & glycerol with biodegradable polymer such as: (poly butylene adipate-co-terephthalate) (PBAT), manufactured by Basf (Germany) under the commercial name of Ecoflex ® 7011; for black films, the pigment Sicopal Black ® K0095, also from Basf (Germany)	*Mechanical properties:* tensile properties (tensile strength, tensile modulus, & elongation) *Biodegradation:* weight loss *Cultivation:* plant dry mass	The biodegradable bags remained intact throughout the seedling production period (60 days) However, after being transplanted into containers (240 days), the bags were completely biodegraded	^14^
Plant fibers	Biodegradable polyester	*Mechanical properties:* maximum tensile stress & elongation at break *Biodegradation:* degradation index *Cultivation:* plant height, fresh & dry weight of plant, number of inflorescences, distance between bracts, dry matter & fresh weight of stems, bracts/leaves ratio, and morphological and colorimetric traits of plant, bracts, and leaves (color coordinates L*, a*, & b*, chroma C*_ab_ and Hua angle H_ab_)	Increasing the fiber percentage from 10 to 20% resulted in an increment in the degradation index from 1.8 to 2.8, along with a decrement in the tensile strength from 20.2 and 19.8 (for new pots) to 4.5 and 3.7 N.mm^−2^ (for used pots after one year), respectively Colorimetric measurements remained relatively stable	^15^
Paper fiber Lignin	Bio-based polymers: Polylactic acid (PLA), Polyhydroxy-alkenoate (PHA), Soy protein polymer, Polyurethane (PUR), Polyamide (PA)	*Biodegradation:* % weight loss for six months *Cultivation:* plant health & dry weight	The optimal blend was for PLA The biodegradable pots are classified into three categories based on degradability: degradable containers in soil within one to two years, compostable containers, and durable containers	^16^
Newspaper pulp fibers	Tapioca (cassava) starch, vinegar, & glycerol	*Physical properties:* water absorption *Mechanical properties:* tensile & puncture strength *Biodegradation:* weight loss of pots	The weight loss of bioplastic pots tested below ground is higher than that of those tested above ground bioplastic pots composed of 75% starch and 25% newspaper fibers exhibited the highest weight loss (77.93%) when tested below ground, and a weight loss of 46.93% when tested above ground The presence of the fungus Deuteromycetes on bioplastic pots may contribute to the degradation process	^17,18^
Residual substrate of medicinal mushroom G. lucidum leaves: [- Sunflower Seed Hull-based Ganocetas - Riceagro-based residue Ganocetas.]	Mycelium net	*Physical properties:* apparent density, effective pore space, & water and air porosity *Mechanical properties:* tensile and flexural strength *Chemical properties:* pH, EC, C/N ratio & chemical contents *Cultivation:* germination and seedling growth or vigor of 17 plant species, & tomato seedling evaluation	The containers offer an advantage: their mycelium cell walls, composed of polysaccharides such as chitin and chitosan, are initially hydrophobic but gradually become hygroscopic while retaining the container structure Additionally, a C/N ratio lower than 30 in the pot signifies an advanced stage of biodegradation	^19^
ـــ	Polylactic acid (PLA) extracted from (Bioplast GS 2189) & starch	*Chemical properties:* thermogravimetric analysis (TGA), Molecular Weight (Mw) determination, Differential Scanning Calorimetry analysis (DSC), & Polymers Identification by identiPol	Mw of PLA after pot processing reduced from 55000 g/mol to 46,300 g/mol Crystallinity increased from 62.4% to 69.9% Injection processing inducing degradation due to the shear forces and thermal variations	^20^
Biosolids from wastewater & fibrous materials (cardboard or Cellulose)	Starch Polymer [FLOPAM 4550 (SNF Inc.)] Natural glue	*Mechanical properties:* tensile strength *Chemical & biological properties:* Saturated Paste Test, pH, EC, leachate elements of pot & algal/fungal growth *Cultivation:* germination, media capacity, resiliency & leachability of pots, & growth and plant health	The material of the container did not impact the concentrations of nutrients or metals in the leachate	^21^
Protein hydrolysate (Pr.Hyd) from leather industry wastes, saw dust & wood flour	poly (ethylene glycol) diglyceryl ether (PEG) or epoxidized soybean oil (ESO), ethylene diamine (EDA)	*Physical properties:* swelling capacity *Mechanical properties:* tensile properties (tensile strength, tensile modulus, & elongation) & Dynamic-mechanical thermal analysis (DMTA) *Biodegradation:* weight loss *Cultivation:* plant height & pepper weight	The biodegradable pots remained intact from seedlings to transplanting, enduring for 35 days before completely degrading within a span of 16 days thereafter	^22^
Straw fiber	Hydrolyzed soy protein isolate/urea/formaldehyde	*Mechanical properties:* tensile strength *Chemical properties:* Fourier-transform infrared spectroscopy (FTIR), differential scanning calorimetry (DSC) curve of the samples, Thermogravimetric analysis (TGA), & X-ray diffraction (XRD) *Morphological properties:* scanning electron microscopy (SEM) *Biodegradation:* % weight loss	Degradability reached 40% after 24 months of soil burial testing due to the rupture of the peptide chains within the composite, resulting in the formation of short amino acids that are readily accessible to microorganisms. This process occurs without causing pollution, in contrast to plastic pots	^23^
*Posidonia oceanica* lignocellulosic fibers (10% of the total weight) derived from “egagropili” (i.e. agglomerates of residues of dead rhizomes and leaves	Thermoplastic matrix PHI002™ consisting of poly(3-hydroxybutyrate-co-3-hydroxyvalerate) (PHBV) a polymer synthesized by a variety of microorganisms, plasticizer acetyl tributyl citrate, and calcium carbonate	*Planting design:* planting in seagrass, dune, and field *Biodegradation:* % weight loss *Cultivation:* total number of alive vs. dead plants, maximum shoot length, mean root length	Biodegradable pots degraded within three years of transplantation seawater and dune sand	^24^
Banana peels	Tapioca starch, vinegar, & glycerol	*Chemical properties:* carbon/nitrogen (C/N) ratio *Biodegradation:* weight loss percentage of pots	Increasing banana peels led to increase in tensile strength consequently, decreasing in decomposition time	^25^
Cellulose fibers (cellulose ProMC^2000^ (ProAgro) with the length of 1350 μm	Metakaolin KM 60 (Keramost), quartz sand, NaOH solution, aluminum powder (Fluca), hydrogen peroxide solution (H_2_O_2_), & mineral solution	*Physical properties:* size distribution, circularity, porosity %, and density *Mechanical properties:* strength by Schmidt hammer *Chemical & biological properties:* pH, X-ray diffraction (XRD), energy dispersive spectroscopy (EDS) analysis *Morphological properties:* scanning electron microscopy (SEM) *Cultivation:* germination and growth of spring wheat, (nitrogen) N, (phosphorous) P, and (potassium) K spectroscopic analysis of leachate	Geopolymer pots retain more water than plastic ones, which can lead to excessive evaporation. This is due to moisture moving from internal to external walls through capillaries in the material. To improve functionality for large-scale production and greenhouse cultivation, additional treatments like painting or impregnation of outer walls are recommended	^26^
Sugarcane bagasse, cornhusk, malt bagasse, & orange bagasse	Cassava starch & glycerol plasticizer	*Physical properties:* thickness, density, & water sorption isotherms *Mechanical properties:* tensile strength & elongation *Chemical properties:* Fourier-transform infrared spectroscopy (FTIR) *Morphological properties:* scanning electron microscopy (SEM) *Biodegradation:* weight loss of trays	Trays containing 20–30% orange bagasse underwent complete degradation within 60 days Trays containing 30% cornhusk had the highest tensile strength (0.57 MPa), whereas the tray containing 30% orange bagasse had the lowest tensile strength	^27^
Alkali treating of nonwoven jute with NaOH	Modified soy resin prepared by: [poly (ethylene glycol), glyoxal, glycerol, soy milk]	*Physical properties:* moisture absorption *Mechanical properties:* tensile & flexural stress, impact testing, microhardness, dynamic mechanical analysis, *Chemical & biological properties:* Fourier-transform infrared spectroscopy (FTIR), thermogravimetric analysis (TGA) & fire retardancy (limiting oxygen index testing) *Morphological properties:* scanning electron microscopy (SEM) *Biodegradation:* soil burial study *Cultivation:* plant root growth	The pots achieved complete biodegradability within 120 days Among all composites, the one containing 60 wt.% nonwoven jute exhibited the highest tensile strength (48.8 MPa), flexural strength (42.5 MPa), and impact strength (15.64 kJ/m^2^)	^28,29^
Lignocellulosic waste materials such as: sawdust, softboard trimming waste, tree bark, coconut fiber, shredded currency notes	Paper mill sludge binder solution	*Physical properties:* water retention *Biodegradation:* biodegradation for ten days *Cultivation:* growth pattern of root	All samples were found to be successful except sawdust During the biodegradability test, the polybags degraded effectively, except for the portion containing coconut fiber. Interestingly, the degraded polybags exhibited potential as a source of manure, aided by the activity of small earthworms	^30^
Sunflower seed Husks Rice husks Yeba mate waste	Gelatin Wheat–waste flour Corn-waste flour Cellulose paper	*Physical properties:* density, solubility, water absorption *Mechanical properties:* tensile and flexural strength *Chemical properties:* carbon/nitrogen (C/N) ratio *Biodegradation:* weight loss *Cultivation:* plant growth percentage	The gelatin-based biocomposite pot exhibited the highest decomposition rate (62%), whereas the other pots demonstrated rates below 28% over a 24-day experiment - Gelatin-based biocomposites are favorable for use in plantable pots, whereas formulations consisting of wheat and corn-waste flour, as well as paper, are prepared for compostable pots	^31^
(Paper substrates newspaper & corrugated cardboard) combined with Textile waste (cotton & polycotton)	Molasses Sodium alginate Cornstarch	*Mechanical properties:* Tensile & compression strength *Biodegradation:* anaerobic degradability, including specific methane and biogas yield, % COD reduction, and % volatile solids decline & % weight loss of pots *Cultivation:* seed germination test	The pots decayed faster than Jiffy pots through a 120-day soil burial test Alkali treatment using 5% NaOH for 5 h increased the ensile strength of the pots	^32,33^
Cattle manure & sawdust	Cornstarch Sheep’s wool	*Physical properties:* water absorption, thickness swelling *Mechanical properties:* rupture load, internal bonding strength *Biodegradation:* decomposition testing *Cultivation:* plant root & shoot height	Pots containing sheep’s wool decomposed in 33 days Cornstarch-based pot has the best- timed degradation and binding	^34^
Paddy straw with alkali and autoclave – treatment & without treatment	Corn starch, boric acid, and glycerol	*Physical properties:* % water uptake, % disintegration in aqueous medium, porosity %, and density *Mechanical properties:* tensile stress & strain *Chemical & biological properties:* Fourier-transform infrared spectroscopy (FTIR), Macro- and micro-nutrients of biocomposites, boric acid leachate & antimicrobial activity *Biodegradation:* CO_2_ emission & weight loss *Cultivation:* mean shoot length, mean root length, fresh weight, dry weight & seedling vigor index (SVI) of cucumber seedling	Biocomposite pots degraded within 10–20 days of transplantation under natural soil circumstances	^35^
Waste oil palm fruit bunches & banana stems with NaOH treatment	Tapioca flour adhesive solution	*Physical properties:* Mass & dimensions, Moisture content, water uptake *Mechanical properties:* tensile strength *Biodegradation:* weight loss percentage	The biodegradability of the pot varied from 40.54 to 76.39%, while the tensile strength ranged from 8091 to 23,418 Pa Uneven fiber dispersion observed in the samples	^36^
Leaf litter	Starch	*Biodegradation:* decomposition rate & CO_2_ efflux for both soil-only incubation and plant-incubated conditions *Chemical & biological properties:* Microbial activity (five soil extracellular enzyme activity: *CBH*: cellobiohydrolase; *BG*: β -1,4-glucosidase; *BX*: β -1,4- xylosidase; *NAG*: β-1,4-N acetylglucosaminidase; *AP*: acid phosphatase) & inorganic nitrogen availability	Recycled waste leaf litter pots exhibited high decomposition rates within 92 days, as well as significant CO_2_ efflux The decrease in inorganic nitrogen content was observed in the presence of plants, suggesting enhanced nutrient availability for both plants and microbes Plant root physiological activity did not directly promote decomposition; however, it resulted in the reduction of soil nutrient content due to nutrient absorption by the plants	^37^
Soy hulls	Soy protein isolate (SPI) with polylactic acid (PLA) matrix	*Biodegradation:* % weight loss *Cultivation:* height, width, shoot fresh weight, fresh root weight, shoot dry weight, and root dry weight	The formulation of biodegradable pots was found to be successful in terms of plant growth and biodegradability	^38^

## Materials and methods

### Raw materials and chemicals

Sugarcane bagasse was used as the primary lignocellulosic reinforcement material and was sourced locally. The raw bagasse was thoroughly washed with distilled water to remove adhering impurities, oven-dried at 60 °C until constant weight, and mechanically ground into a fine powder.

Auxiliary fillers, including compost, peat moss, vermiculite, and activated carbon, were obtained from local agricultural suppliers and used without further chemical modification.

Palm wax and Lanette wax (hexadecanol-based) were employed as biodegradable binding matrices. Sorbitol was used as a plasticizer to enhance flexibility. Sodium hydroxide (NaOH, analytical grade) was used for alkaline pretreatment (mercerization) of the fibers.

### Alkaline pretreatment of bagasse fibers

Alkaline treatment was performed to improve fiber–matrix interfacial bonding by partially removing lignin and hemicellulose while preserving the cellulose structure.

Bagasse fibers were immersed in a 1 M NaOH solution at 60 °C for 30 min under mild pressure conditions of about 1.5 kPa. This treatment condition was selected based on previous studies demonstrating effective delignification without significant degradation of cellulose chains^39^.

Following treatment, the fibers were thoroughly rinsed with distilled water until the pH was neutral to remove residual alkali. The treated fibers were then dried using microwave irradiation at 600 W until constant weight.

### Preparation of biocomposite formulations

Biocomposites were prepared by blending 35 wt % sugarcane bagasse (treated or untreated) with wax matrices at a fixed weight ratio of 4:5 (fiber:wax). Auxiliary fillers (35 wt % compost, 10 wt % peat moss, 10 wt % vermiculite, and 10 wt % activated carbon) were incorporated in minor proportions to enhance structural and functional properties (qualitative enhancement rather than primary load-bearing contribution).

The mixture was heated to the melting temperature of the respective wax (60 °C), followed by continuous manual mixing to ensure homogeneous dispersion of fibers within the matrix.

The molten composite was then poured into steel molds and subjected to hot pressing at 160 bar and 60 °C to form the final cultivating pots.

### Classification of fabricated pots

The fabricated pots were categorized based on binder type and fiber treatment as follows:

- *P-Pot*: Palm wax + untreated bagasse.
- *PW-Pot*: Palm wax + NaOH-treated bagasse.
- *L-Pot*: Lanette wax + untreated bagasse.
- *LW-Pot*: Lanette wax + NaOH-treated bagasse.

### Chemical properties of cultivating pots:

#### pH and electrical conductivity (EC):

A saturated aqueous composite extract 1:5 mass ratio of grinding composites to water suspension from each mix constituent, which was soaked at room temperature for 24 h incubation, was extracted for measuring the properties by the potentiometric method using a pH and EC meter. The leachate extract was filtered with a mesh cloth before measuring^40^.

#### Elemental composition analysis of the cultivating pots:

Chemical elemental analysis was performed using scanning electron microscopy equipped with energy-dispersive X-ray spectroscopy (SEM–EDX). Pre-cut samples (1 cm × 1 cm) were analyzed at an accelerating voltage of 20 kV.

The technique was used to determine the relative abundance of major elements (C, N, O) and of essential macro- and micronutrients within the composite matrix.

#### Attenuated total reflectance fourier transform infrared (ATR-FTIR) spectra analysis of cultivating pots:

Functional group characterization was carried out using Attenuated Total Reflectance Fourier Transform Infrared (ATR-FTIR) spectroscopy (THERMO NICLOT ATR-FTIR-50).

Spectra were recorded in the range of 4000–500 cm⁻^1^ to identify chemical modifications associated with alkaline treatment, particularly changes in lignin and hemicellulose-related functional groups^41^.

#### X-ray diffraction (XRD) analysis for cultivating pots:

The X-ray diffraction profile pattern for the cultivating pots was measured using a Bruker D2 phaser, 2^nd^ generation, X-ray diffractometer, ranging from 5 to 80, with 32 times per step, operating at 40 kV, 20 mA, and CuKα radiation scan type (λ = 1.5406 Å). After performing baseline alignment and subtraction using OriginPro 2018.

### Morphological properties of cultivating pots:

#### Qualification of micrographs for cultivating pots (SEM imaging)

Surface morphology of the composites was examined using a field emission scanning electron microscope (FEI Quanta FEG 250) at an accelerating voltage of 20 kV. Samples were prepared as 1 cm × 1 cm sections and analysed to evaluate fiber dispersion, interfacial bonding, and structural compactness.

#### Quantification of micrographs for cultivating pots (ImageJ-based quantitative analysis)

Quantitative analysis of SEM micrographs was performed using ImageJ software to evaluate microstructural parameters, including:

- Homogeneity coefficient (ζ)
- Deviation rate (η)
- Surface porosity
- Surface profile topography (Surface roughness and waviness)
- Grain intensity distribution
- 2-D fast Fourier transformation

To ensure reproducibility, all SEM images were acquired under identical conditions, and analysis parameters were standardized across all samples. Each measurement was conducted in triplicate, and mean values were reported.

#### Surface porosity analysis

Surface porosity was quantified using an image processing approach based on pseudo-color segmentation and intensity thresholding.

SEM images were processed in ImageJ by applying a color mapping scheme to enhance contrast between solid and low-intensity regions. Thresholding was performed to isolate pore regions corresponding to low-intensity areas.

Binary masks were generated to distinguish pores from the solid matrix, and porosity (%) was calculated as:

$${\mathrm{Porosity}} = \frac{{{\mathrm{Void}}\;{\mathrm{Area}}}}{{{\mathrm{Total}}\,{\mathrm{Area}}}} \times 100$$

To ensure consistency, identical threshold criteria were applied across all samples. Although pseudo-color mapping was used for enhancement visualization, segmentation was based on underlying intensity differences, consistent with conventional grayscale thresholding approaches.

#### Homogeneity analysis

Microstructural homogeneity was evaluated using intensity-based statistical parameters:

$$\zeta = \left[ {1\frac{{{\mathrm{STD}}\left( {A_{i} } \right)}}{{E\left( {A_{i} } \right)}}} \right] \times 100$$

$$\eta = \frac{{{\mathrm{max}}\left( {A_{i} } \right) - {\mathrm{min}}\left( {A_{i} } \right)}}{{E\left( {A_{i} } \right)}} \times 100$$

where: E (Ai) represents the main value of intensity, STD (Ai) represents the standard deviation of intensity, min (Ai) represents the minimum value of intensity, and max (Ai) represents the maximum value of intensity^42^.

#### Surface topography and roughness

**Fig. 1:**
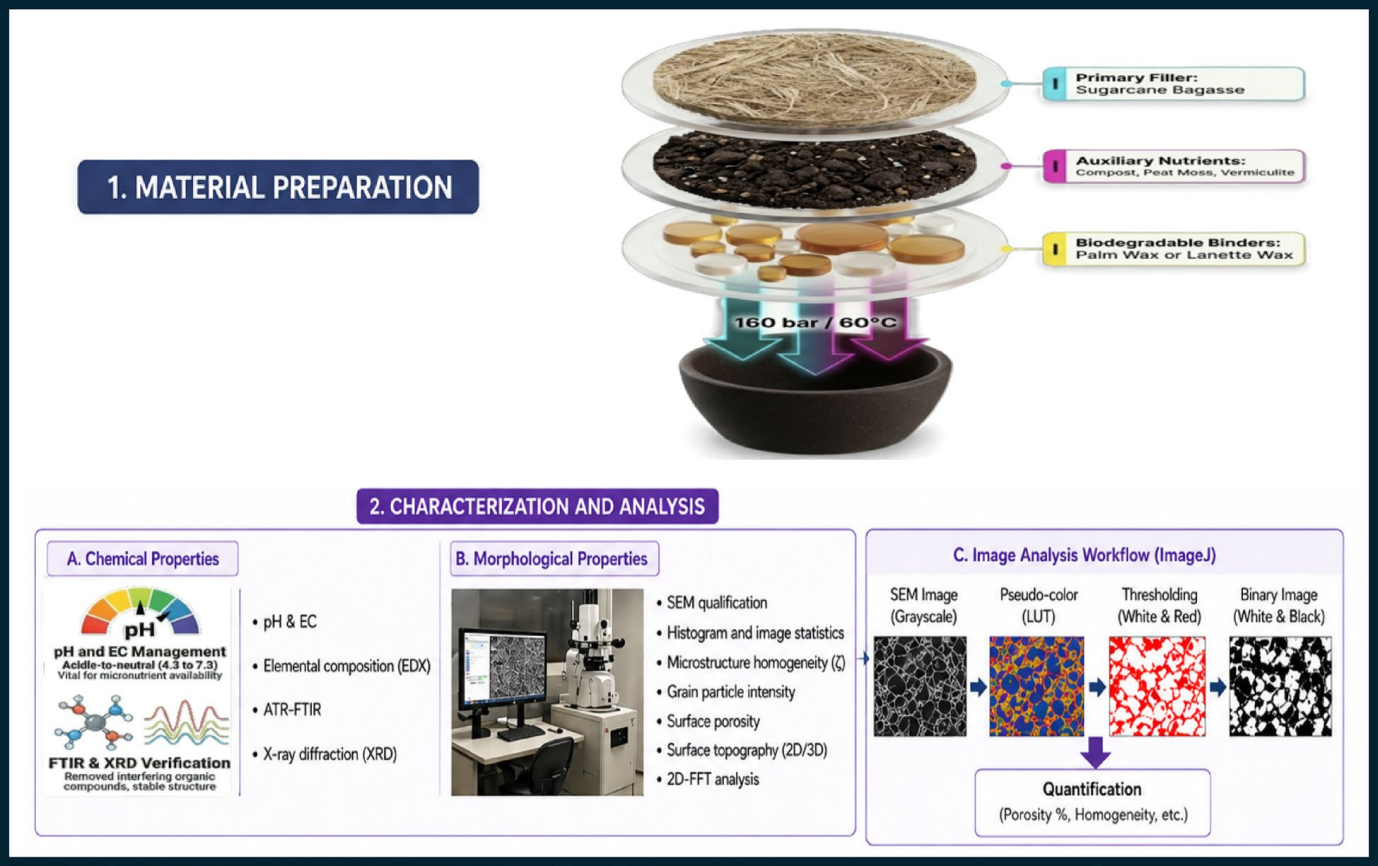
Schematic representation of the experimental workflow for the development and characterization of biodegradable biocomposite pots. (1) Material preparation involves the formulation of sugarcane bagasse as the primary filler, combined with auxiliary nutrients (compost, peat moss, vermiculite) and biodegradable binders (palm or lanette wax), followed by thermal pressing under controlled conditions. (2) Characterization includes evaluation of chemical properties (pH, EC, elemental composition via EDX, ATR-FTIR, and XRD) and morphological properties using SEM analysis, image statistics, and microstructural assessment. (3) Image analysis workflow using ImageJ consists of grayscale SEM imaging, pseudo-color mapping via look-up table (LUT) for enhanced visualization, intensity-based thresholding (white/red segmentation), and binary image conversion (black/white) for quantitative analysis of porosity and structural homogeneity.

Surface topography was analyzed using ImageJ throughout Gaussian filtering to separate waviness and roughness components. Three-dimensional surface plots were generated, and roughness values were expressed as mean ± standard deviation. The overall experimental design and analysis workflow are illustrated in Fig. 1.

#### Statistical analysis of image data

Statistical fitting and regression analysis of grain intensity distributions were performed using OriginPro software. Normal distribution fitting was applied, and regression coefficients (R^2^) were used to evaluate the consistency of structural patterns. Statistical differences between samples were evaluated using one-way ANOVA at *p* < 0.05.

## Results and discussion

### Chemical properties of cultivating pots:

#### pH and EC measurement of cultivating pots

The pH plays a crucial role in plant soil, as it significantly influences the availability of micronutrients in the growing medium. At high pH levels, certain nutrients essential for optimal plant growth become unavailable, leading to the development of deficiency symptoms in plants^43^. Understanding and effectively managing pH and electrical conductivity (EC) are vital components of sustainable agriculture. Regular monitoring, appropriate amendments, consideration of osmotic potential, and influencing water ion uptake by plant roots, coupled with the implementation of suitable cultivation practices, contribute to optimizing healthy plant growth and preserving soil health.

All the pots exhibited acidity, ranging between 4.3 and 6.8, except for PW and V-pots, which showed alkaline properties with values of 7.3 and 7.1, respectively. It is well-known that the pretreatment with alkaline sodium hydroxide tends to elevate pH, as depicted in Table 2. The highest EC was recorded for C-pot at 397 ppm, while the lowest was observed for V-pot at 151 ppm. This outcome aligns with findings from^44^.

**Table 2:** pH and EC measurements of cultivating pots are made from different fillers.

Filler type	pH	EC (ppm)
P-pot	6.7	346
PW-pot	7.3	359
L-pot	5.4	220
LW-pot	5.9	205

#### Chemical elemental composition analysis of cultivating pots:

**Fig. 2:**
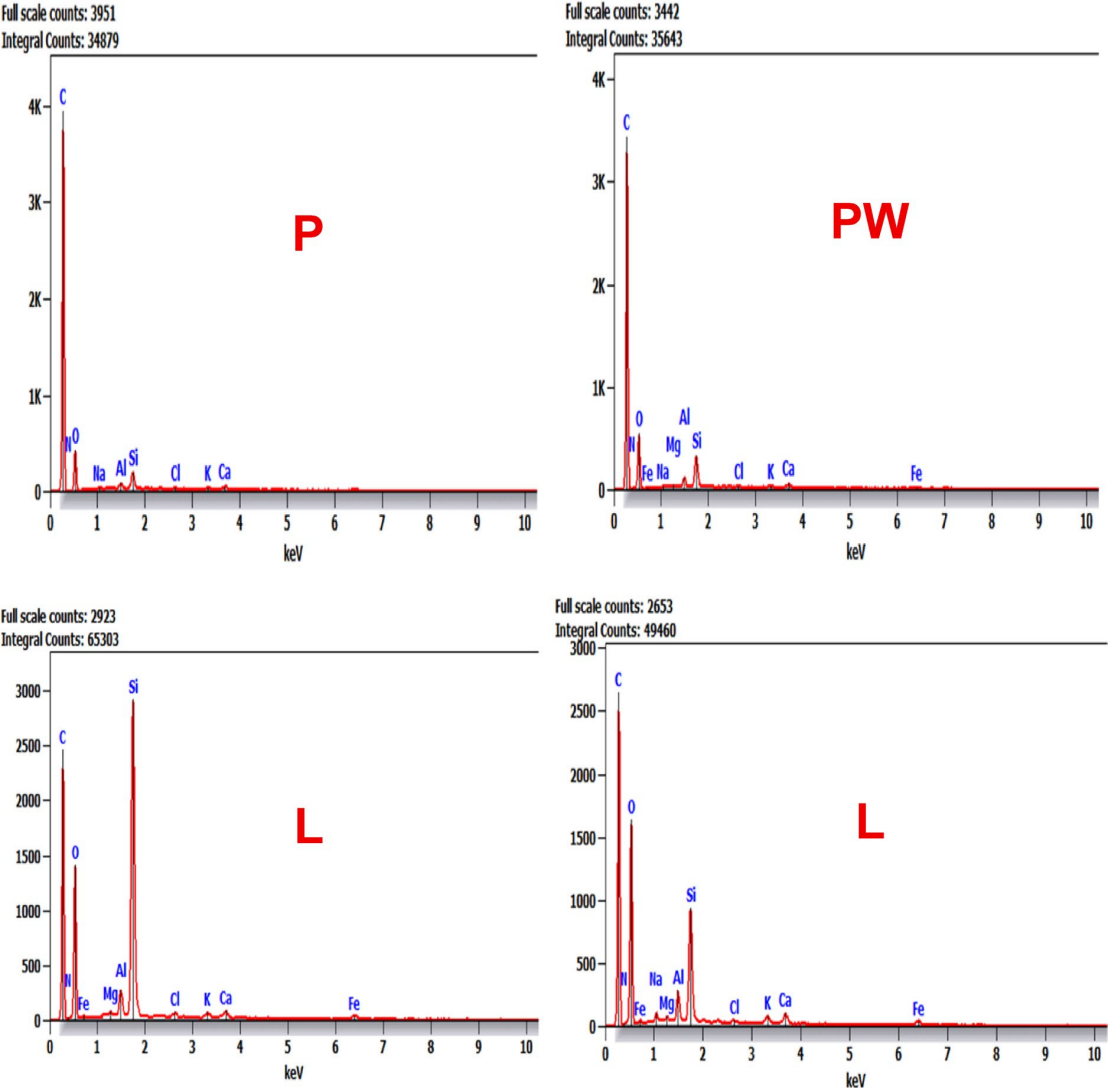
Energy-dispersive X-ray diffraction (EDX) spectrum of palm wax and hexadecanol-based pots.

Energy Dispersive X-ray Diffraction (EDX) coupled with Scanning Electron Microscopy (SEM) was employed for the elemental analysis of cultivating pots. The EDX spectrum, shown in Fig. 2, revealed peaks corresponding to the binding energies of carbon, nitrogen, and oxygen, as well as other macro and micronutrients. In P, PW, L, and L-pot, carbon comprised 54.4%, 49.3%, 37%, and 34.5%, respectively; nitrogen accounted for 11.4%, 10.3%, 5.8%, and 5.3%, respectively, and oxygen constituted 28.3%, 30.3%, 31.4%, and 42.7%, respectively, as indicated in Table 3. Additionally, Mg and Fe elements were detected in P and PW-pots, while the P element coincidentally appeared in L and LW-pots.

**Table 3:** Elemental composition of palm wax-based pots obtained by EDX.

Element	P (weight %)	PW (weight %)	L (weight %)	LW (weight %)
C	54.4	49.3	37	34.5
N	11.4	10.3	5.8	5.3
O	28.3	30.3	31.4	42.7
Na	–	0.4	–	1.2
Mg	–	0.2	0.3	0.4
Al	0.8	1.1	1.5	1.9
Si	2.9	4.4	20.2	8.4
P	–	–	–	0.2
Cl	0.4	0.6	0.6	0.4
K	0.6	0.7	0.7	1.1
Ca	1.1	1.2	1	1.6
Fe	–	1.6	1.6	2.2

It’s important to note that both palm wax and hexadecanol have the main elements of hydrocarbons—carbon, hydrogen, and oxygen—in different molecular formulas. The arbitrary sampling and the composite’s heterogeneity, featuring discontinuous, randomly oriented fibers with different compounds prepared using various methods, may explain the observed differences. For instance, compost and peat moss may contain various nutrients during their preparation. However, there is a limitation in quantification: the samples may contain hydrogen, which SEM cannot detect. Further analysis using Inductively Coupled Plasma Mass Spectrometry (ICP-MS) for quantification from different parts may offer more precise and comprehensive results.

#### Attenuated total reflectance fourier transform infrared (ATR-FTIR) spectra analysis of cultivating pots:

**Fig. 3:**
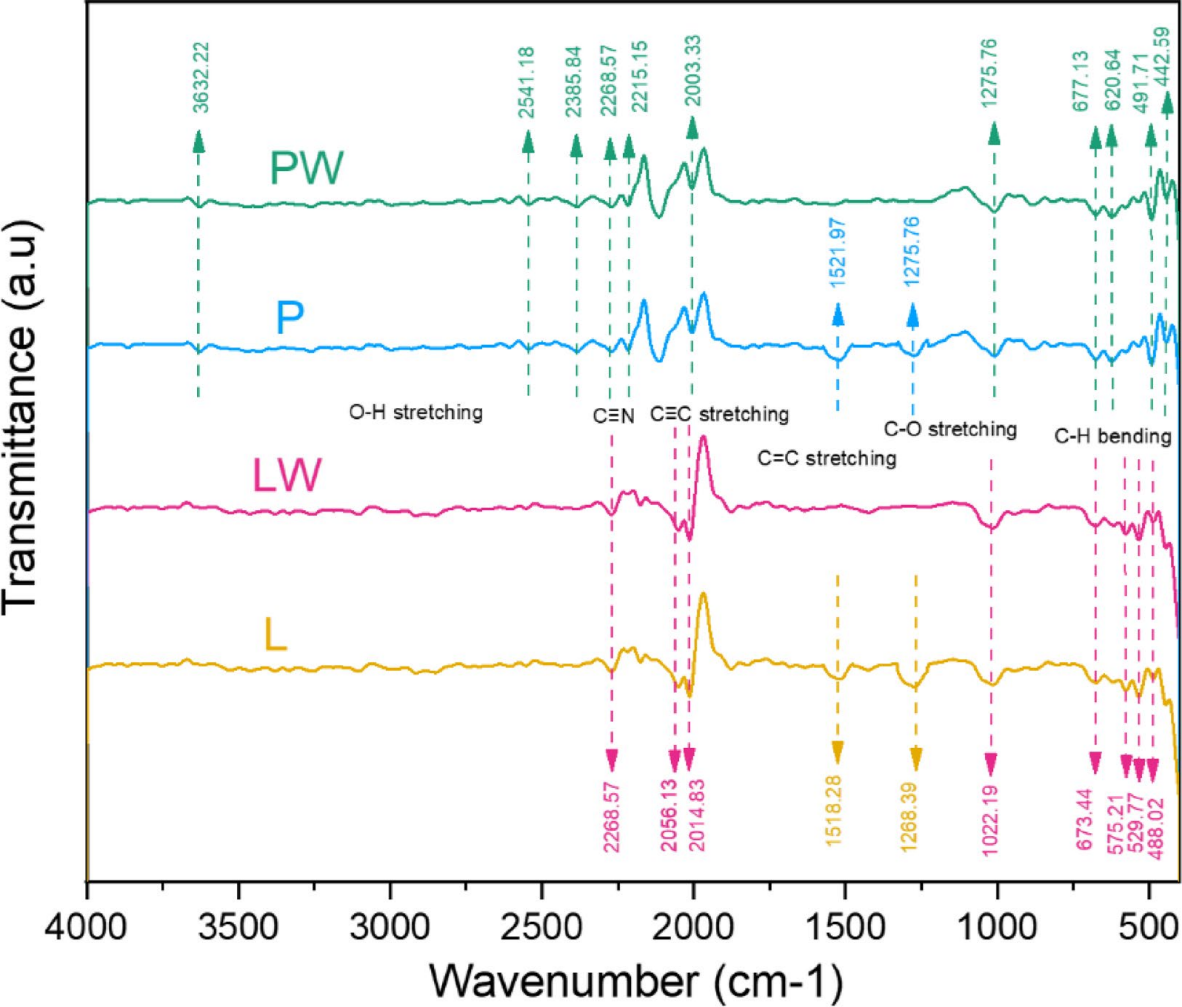
FTIR-ATR transmittance spectra of cultivating pots.

The FTIR-ATR transmittance spectrum of the P-pot, illustrated in Fig. 3, exhibits discernible peaks. A weak sharp peak at 3632.22 cm^−1^ may indicate the presence of OH stretching vibrations from hydroxyl groups in sugarcane bagasse cellulose. The high wavenumber at 2541 cm^−1^ may suggest unique functional groups in activated carbon or the compost material’s structure, possibly involving multiple bonds or highly strained molecular structures. Peak at 2385.84 cm^−1^ could indicate specific functional groups in the vermiculite or even in compost, possibly related to multiple bonds or specific mineral compositions. 2268.57 cm^−1^ peak often corresponds to carbon dioxide (CO_2_) or nitrile (C≡N) stretching vibrations associated with the decomposition products in compost or the presence of carbonates in vermiculite, and peaks at 2056 and 2215 (C≡C) stretching of alkynes. Peaks in 2014 and 2003 cm^-1^ indicated the presence of nitrogen-based, like N=C=S stretching, isocyanate functional group.

The peaks at the fingerprint region 1006 cm^-1^ might be associated with C–O stretching vibrations in the palm wax or other oxygen-containing functional groups. While peaks from 673 to 442 cm^−1^ may indicate C–H bending vibrations in alkanes, alkynes, or aromatic compounds, they are attributed to characteristic interactions or molecular structures of organic ingredients resulting from the combined components in the mixtures.

The presence of peaks from 1525 to 1265 cm^−1^ in the untreated bagasse suggests the combined contributions of lignin and hemicellulose within the sugarcane bagasse. These peaks are attributed to the C=C stretching of the aromatic guaiacyl (G) ring breathing, indicating the richness of lignin in aromatic rings. Additionally, C–O stretching vibrations are present in ester functional groups within polysaccharides existing in lignin and hemicellulose^45^. These analyses are associated with the findings of^41,46–49^.

#### X-ray diffraction (XRD) analysis

X-ray Diffraction (XRD) was conducted to examine the impact of blending various proportions of distinct materials on the diffraction characteristic patterns of the produced cultivating pots and to distinguish between them.

**Fig. 4:**
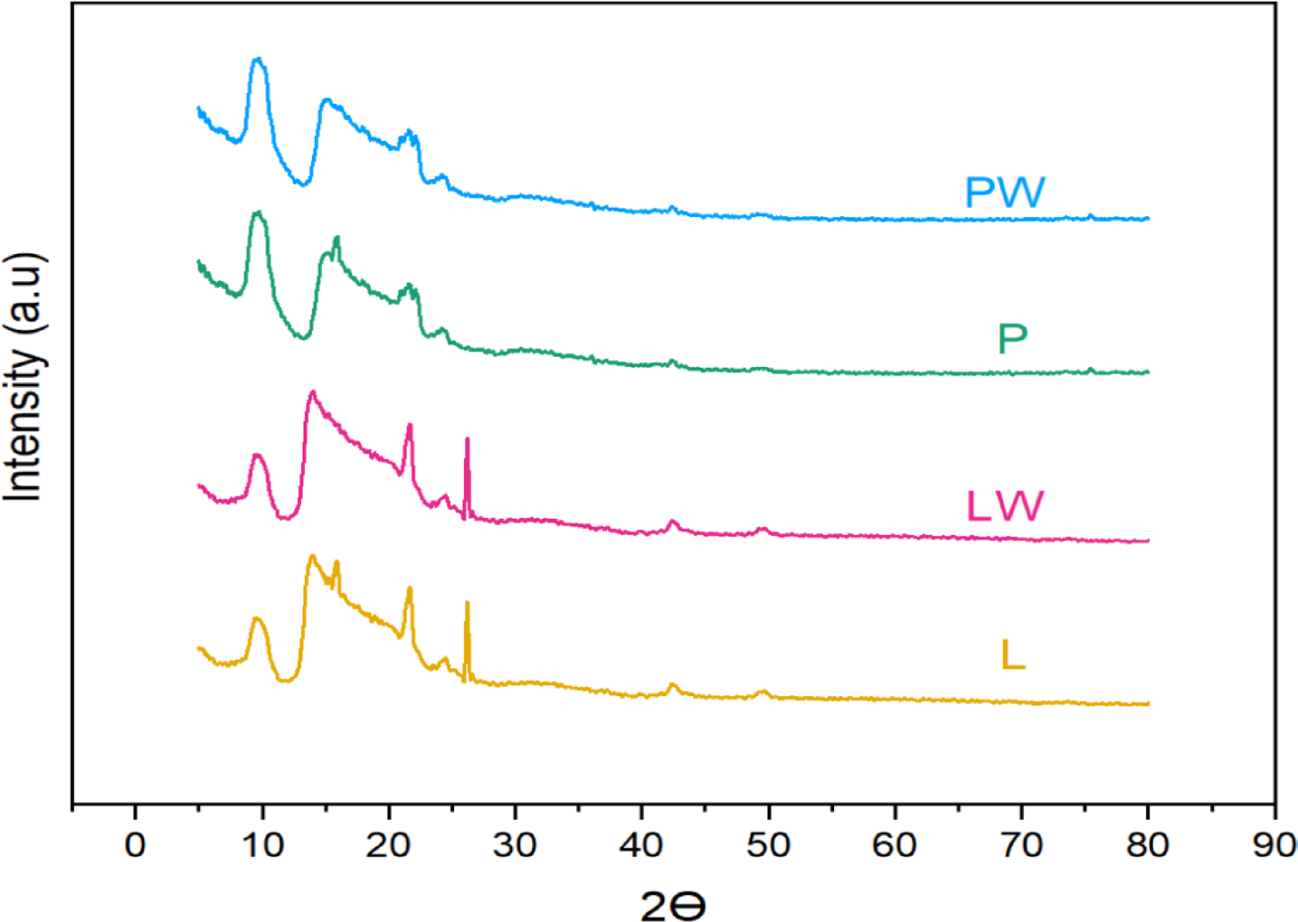
XRD diffractogram pattern of the fabricated cultivating pots.

Figure 4 depicts the X-ray Diffraction (XRD) pattern of amorphous and crystalline regions for the fabricated cultivating pots. The highest diffraction peak was revealed at around 2Ɵ = 9.7°, 9.74°, 13.78°, and 13.78° with corresponding heights of 270.08, 270.4, 220.8, and 222.08 counts, for P, PW, L, and LW, respectively.

The peak at around 2Ɵ of 21.81° is associated with lignin and hardwood^50^, affirming the fiber reinforcement of sugarcane bagasse without pretreatment. The broad peak, commencing at 2Ɵ of 9.74°, shifted earlier than the reference by^51^, which was at 15.6°, and concluded at 24.35°, slightly later than 22°. This shift indicates the influence of mixing different materials in the composite preparation on the typical profile structure.

The peaks at 15.87° and 15.89° for P and L, respectively, disappeared, possibly indicating partial removal of lignin and hemicellulose after mercerization.

The diffractogram for L and LW had a distinct, strong, and sharpest peak at 26.2°, which may indicate the presence of minerals such as phosphorus, as confirmed by EDX.

### Morphological properties of cultivating pots

Morphological characteristics play a crucial role in the final strength of the composite^52^. Therefore, the qualification and quantification of the scanned micrographs have been performed for meticulous distinction among images, and image statistics were also analyzed.

#### Analysis and qualification of SEM images

**Fig. 5:**
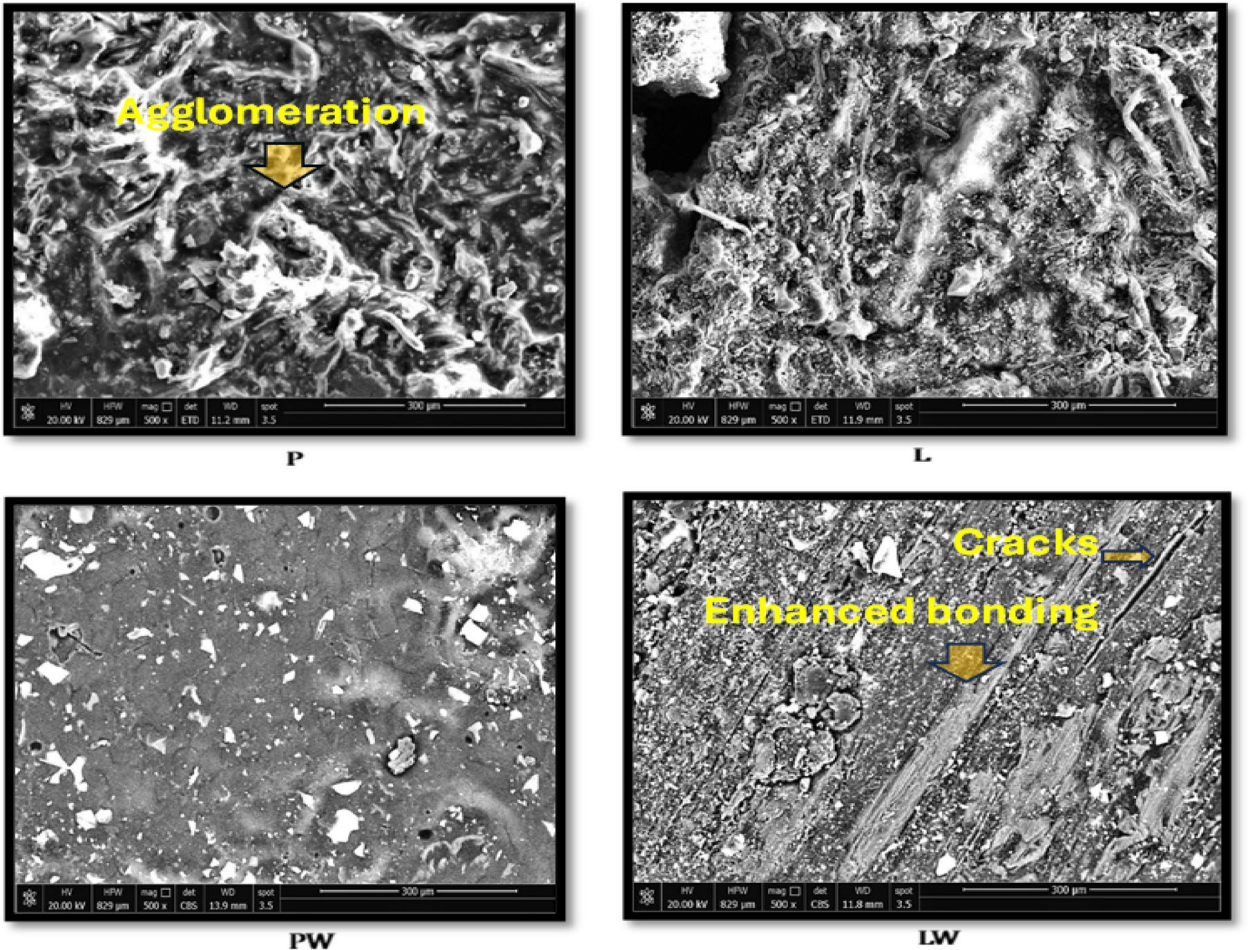
SEM micrographs of the cultivating pots.

In the micrograph images presented in Fig. 5, the composites exhibit a well-compacted and relatively homogeneous structure. However, the presence of cracks may be attributed to a low plasticizer content. It is not recommended to increase the plasticizer, as it can lower tensile strength and has limited compatibility with the adhesive palm wax or hexadecanol, resulting in reduced consistency. The observed variations may also be influenced by using different particle sizes for each filler, each possessing unique characteristics. Additionally, defects in the fabrication process, such as pouring the mats or inherent mat quality, could contribute to these features.

**Fig. 6:**
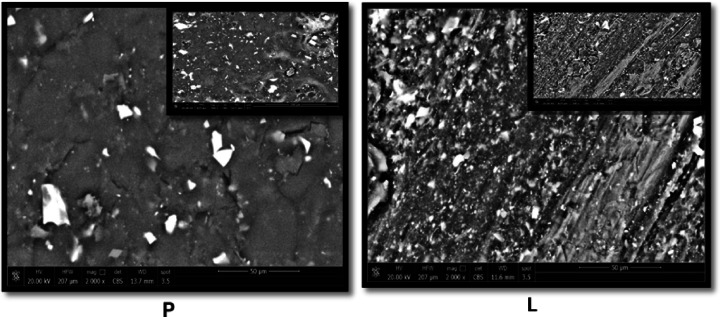
Magnified SEM micrographs of cultivating pots.

Visual inspection suggests a decrease in agglomeration in non-pretreatment samples (P and L-pot) after pretreatment, attributed to surface enhancement of the fibers. Notably, the PW-pot exhibits the best-smoothed, compacted, and regular surface, while the LW-pot demonstrates improved regular distribution post-pretreatment. Further details on enhanced surface properties and compaction micrographs of PW and LW, magnified by 2000, are presented in Fig. 6. These observations align with the tensile properties results of the cultivating pots.

These outcomes are consistent with the observations made by^53^, who noted that the thin-walled fibers of bagasse transform their tubular structure after pressing. This transformation results in an increased surface area conducive to enhanced bonding, ultimately contributing to heightened tensile and burst strength.

#### Histogram and image statistics

**Fig. 7:**
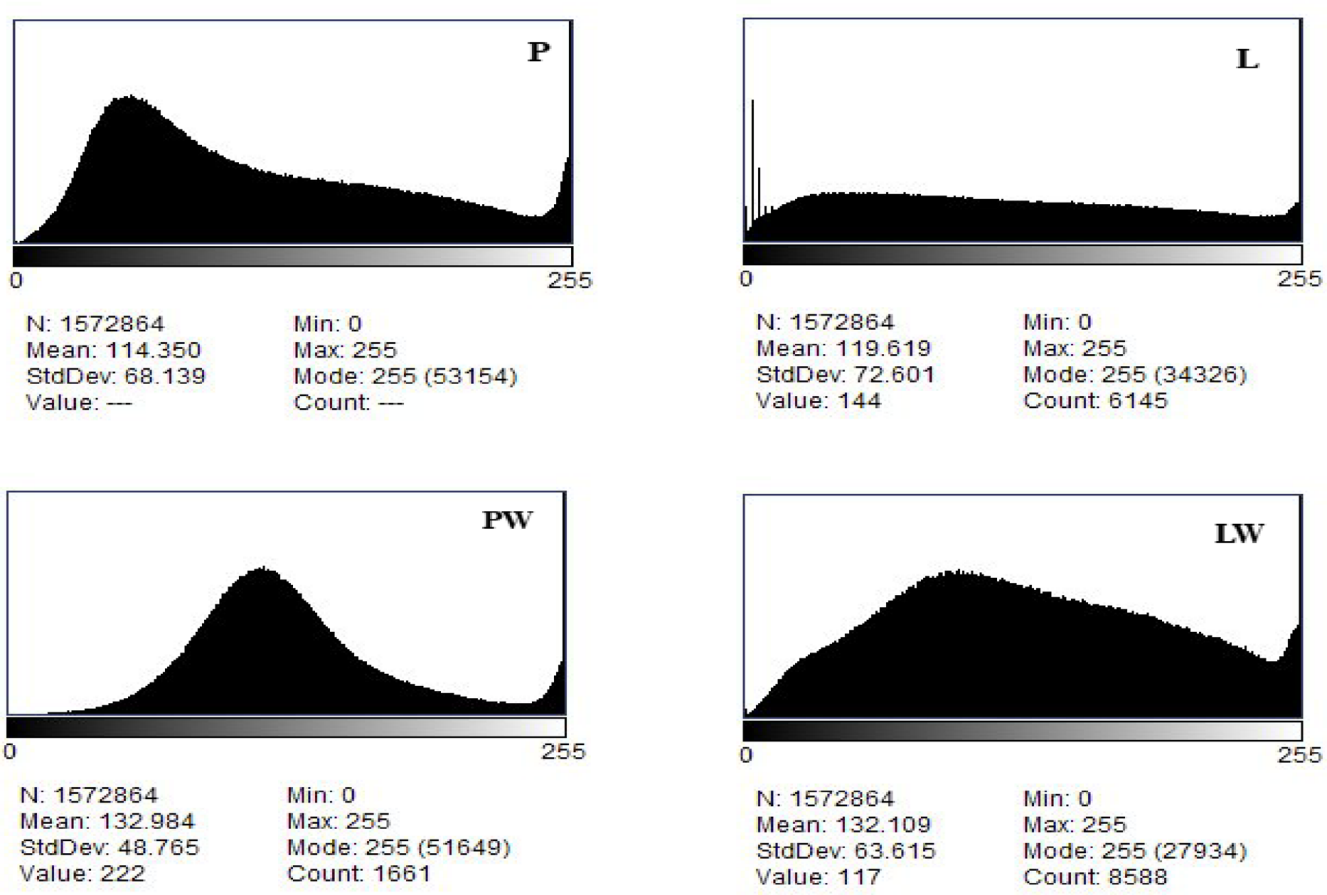
Typical frequency distribution histogram for cultivating pots.

Figure 7 presents grayscale histograms representing the characteristics of cultivating pots. Typically, histograms depict frequency distributions of intensity values, specifically the frequency of each gray value level present within the images. Consequently, they offer a detailed insight into image distribution and aid in identifying potential issues related to image acquisition^54^. Upon examination of the frequency distributions, P, PW, L, and LW exhibit relatively even distributions. Notably, PW demonstrates the most uniform distribution, while the L sample displays solid, sharp lines, indicating outliers and suggesting a less desirable distribution pattern.

#### Evaluation of microstructural homogeneity in cultivating pots

**Fig. 8:**
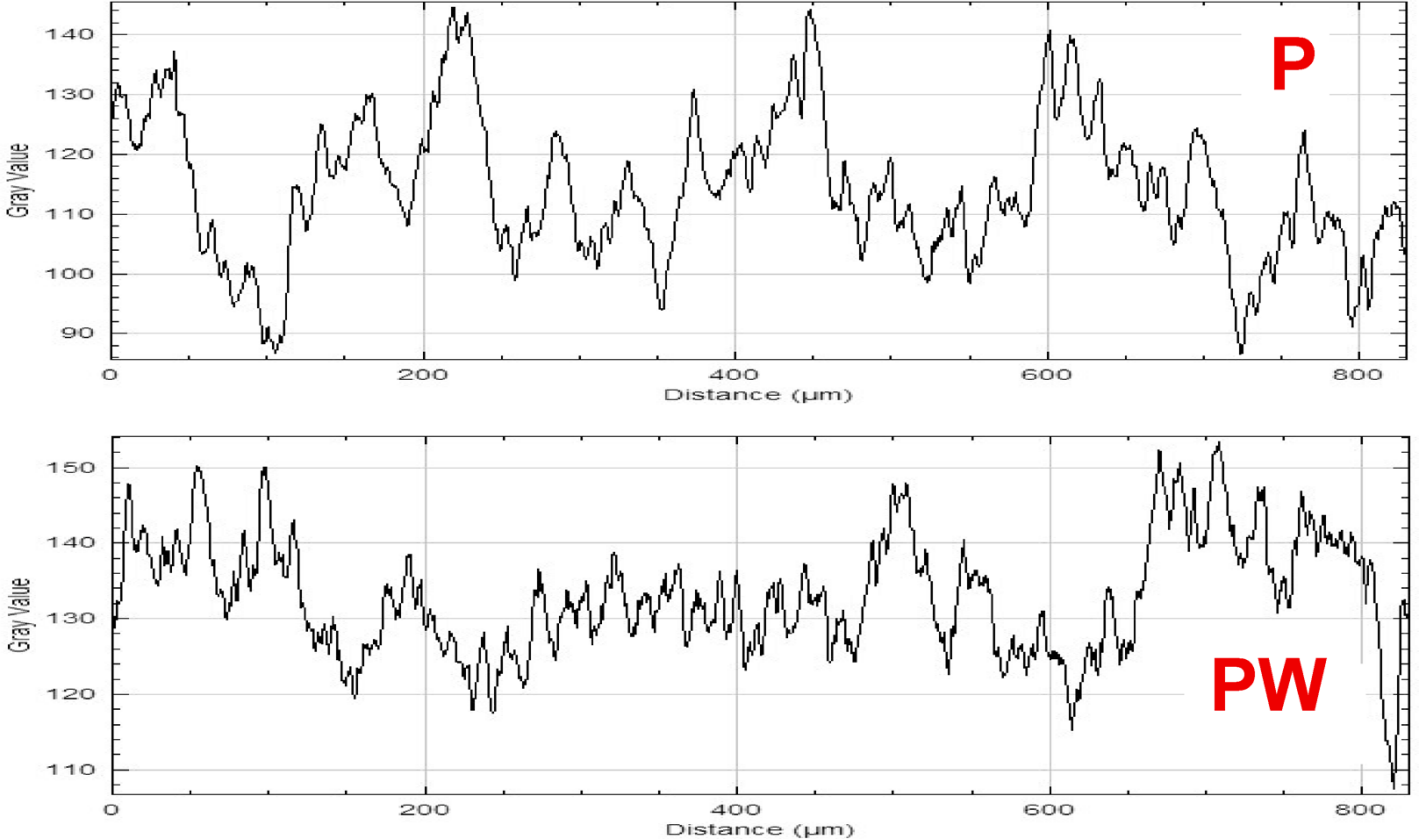
The corresponding surface profile plots of P and PW- pot.

The homogeneity of the reinforcement distribution plays a crucial role in enhancing tensile strength, as supported by^42^. To quantitatively assess the homogeneity of particle distribution and its variation trend, the homogeneity factors for all cultivating pots are presented in Table 4. A higher homogeneity coefficient (ζ) indicates a better distribution index of the composite, while a lower deviation rate (ɳ) is indicative of better homogeneity. Figure 8 illustrates that the standard deviation of the PW-pot is the lowest, consistent with the deviation rate calculated in Table 4.

**Table 4:** Homogeneity factors of cultivating pots.

Label	Homogeneity factors	
	Homogeneity coefficient ζ (%)	Deviation rate ɳ (%)
P	40 ± 5.6^BC^	223 ± 4.6^A^
PW	63 ± 3^A^	192 ± 2^C^
L	39 ± 5.5^C^	213 ± 4.5^B^
LW	51.84 ± 3.7^AB^	193 ± 2.7^C^

Means that do not share a letter differ significantly using one-way ANOVA at *p* < 0.05 (Tukey’s HSD).

The improved homogeneity (ζ = 63%) in PW-pot correlates with reduced porosity (0.43%) and enhanced surface compactness, which likely contributed to improved plant growth performance.

#### Surface profile plots of cultivating pots:

**Fig. 9:**
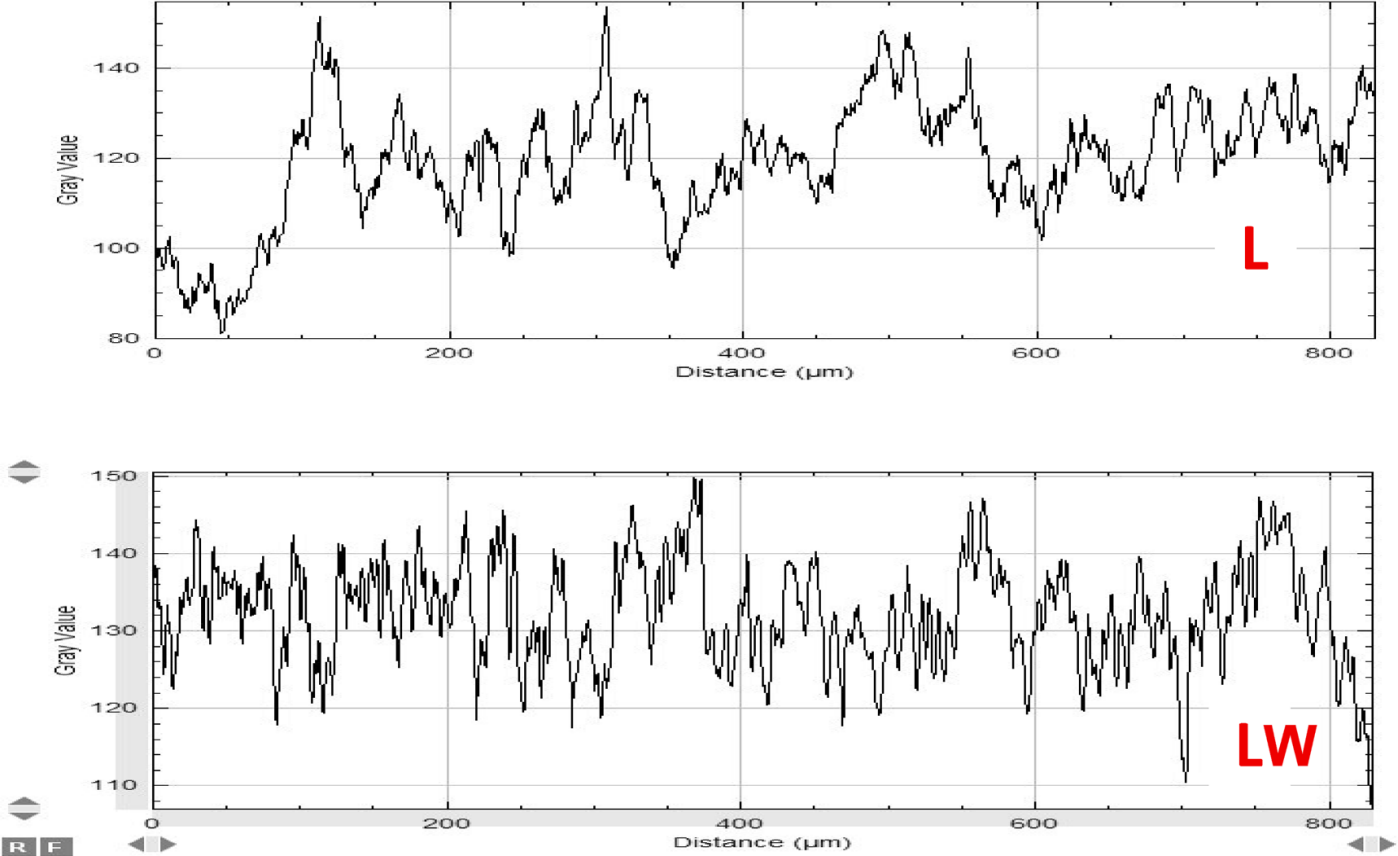
The corresponding surface profile plots of L and LW- pot.

**Fig. 10:**
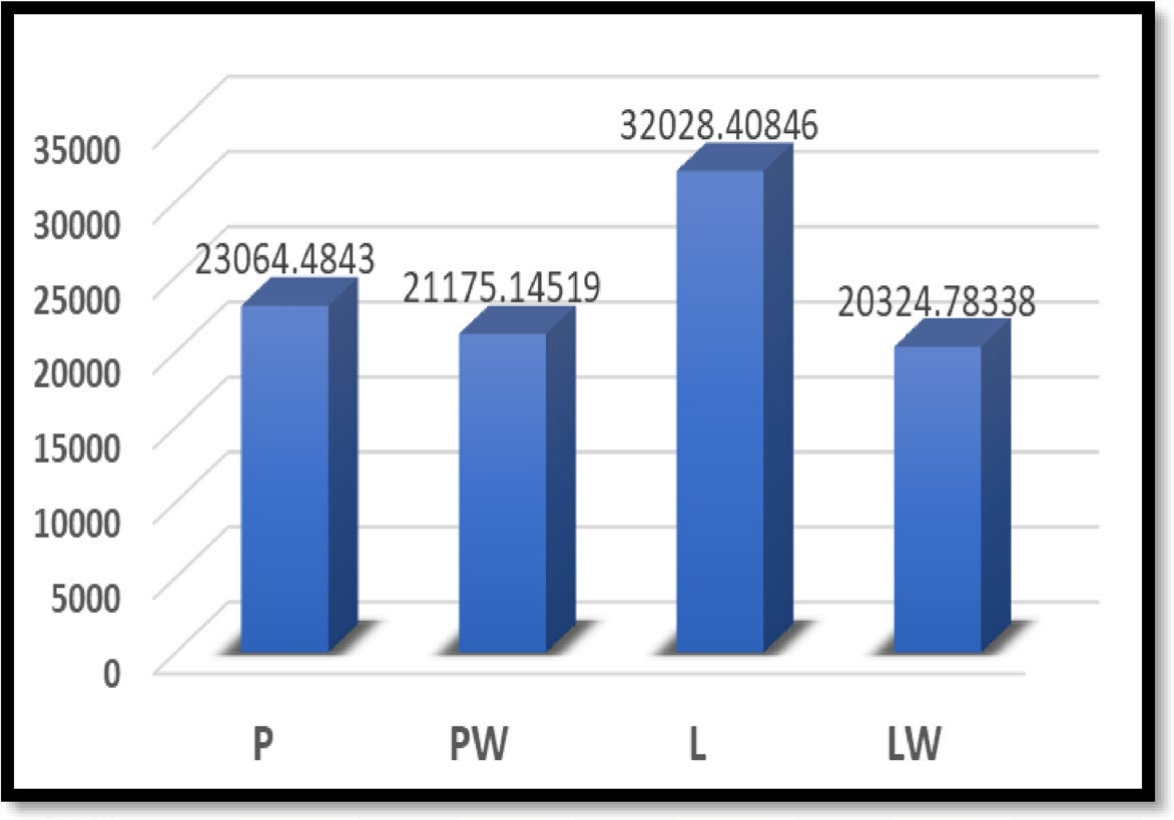
Area integration under the gray value curve of cultivating pots.

The surface profile plots of the cultivating pots generated by ImageJ software, presented with the area under the gray value curve using OriginPro 2018 software in Figs. 8, 9, and 10, offer insights into voids, as mentioned by^54^. The distance between peaks of gray value, i.e., intensity, represents the distance between fibers or voids. Meanwhile, the width of these peaks indicates the grain size or the thickness of different fibers.

#### Statistical evaluation of grain particle intensity:

**Fig. 11:**
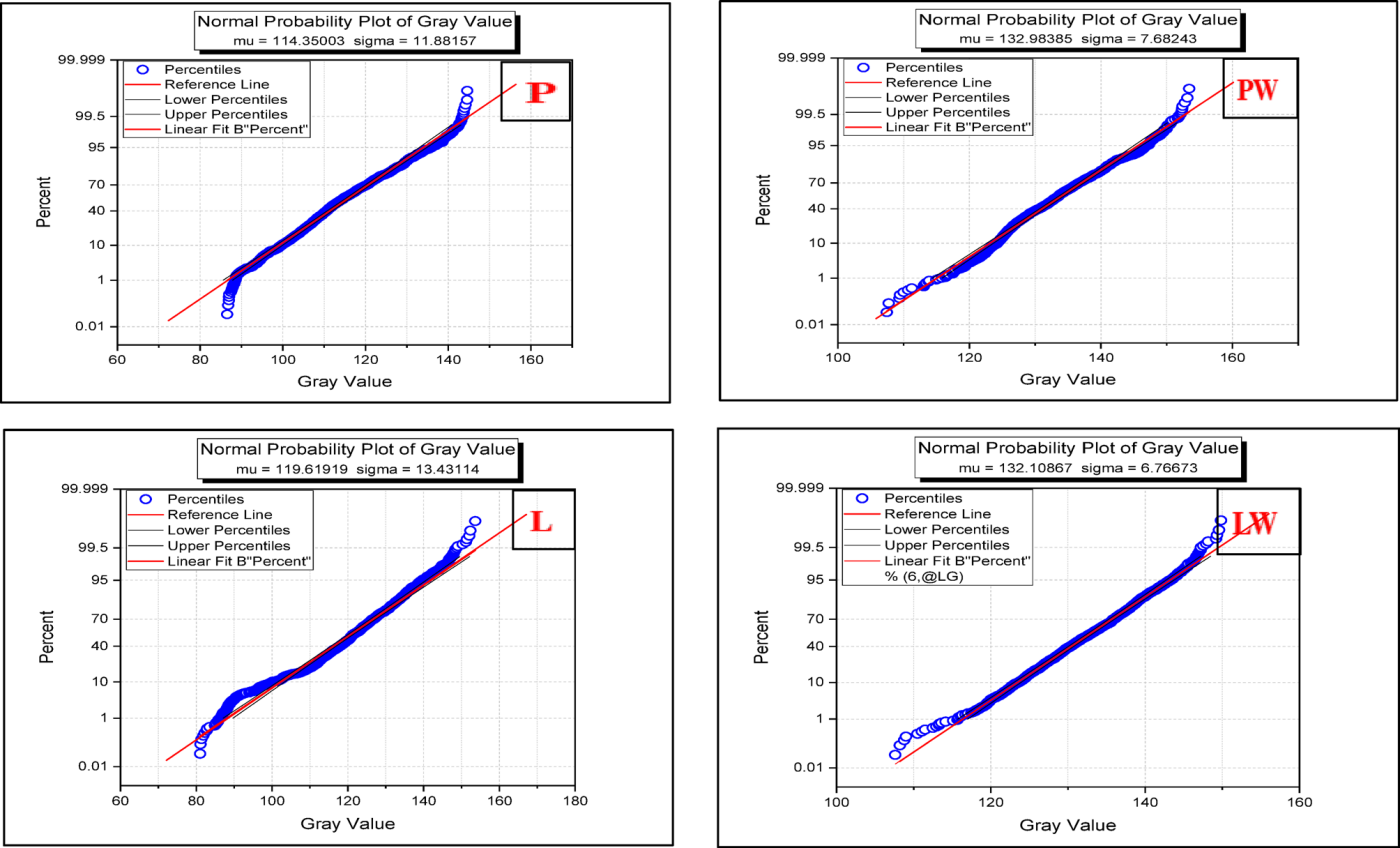
Normal probability plots of P, PW, L, and LW- pot.

For a more in-depth statistical analysis of surface plots and intensity, Origin Pro was employed to calculate the probability distribution of grain particles. This process helps ascertain whether the adhesion of the composite matrix follows the hypothesized distribution or not. Figure 11 illustrates the fitting of the normal distribution of surface plot intensity, indicating linearity. The corresponding regression coefficients and parameter equations can be found in Tables 5 and 6.

**Table 5:** Coefficients for corresponding fitting regression analysis.

Label	Residual Sum of Squares	Pearson’s r	R-Square (COD)	Adj. R-Square
P	11.98503	0.99608	0.99217	0.99217
PW	13.05011	0.99573	0.99148	0.99147
L	30.54507	0.98998	0.98006	0.98004
LW	5.006	0.99836	0.99673	0.99673

**Table 6:** Calculation of slopes and intercepts for regression equations of gray values.

Label	Parameter	Value	Standard error	t-value	Prob >|t|
P	Intercept	− 9.57572	0.02183	− 438.65786	0
	Slope	0.08374	1.90E−04	441.01791	0
PW	Intercept	− 17.21698	0.04082	− 421.78713	0
	Slope	0.12947	3.06E−04	422.4899	0
L	Intercept	− 8.80701	0.03228	− 272.84619	0
	Slope	0.07363	2.68E−04	274.55963	0
LW	Intercept	− 19.46957	0.0285	− 683.05677	0
	Slope	0.14738	2.15E−04	683.95163	0

#### Surface porosity of cultivating pots

**Fig. 12:**
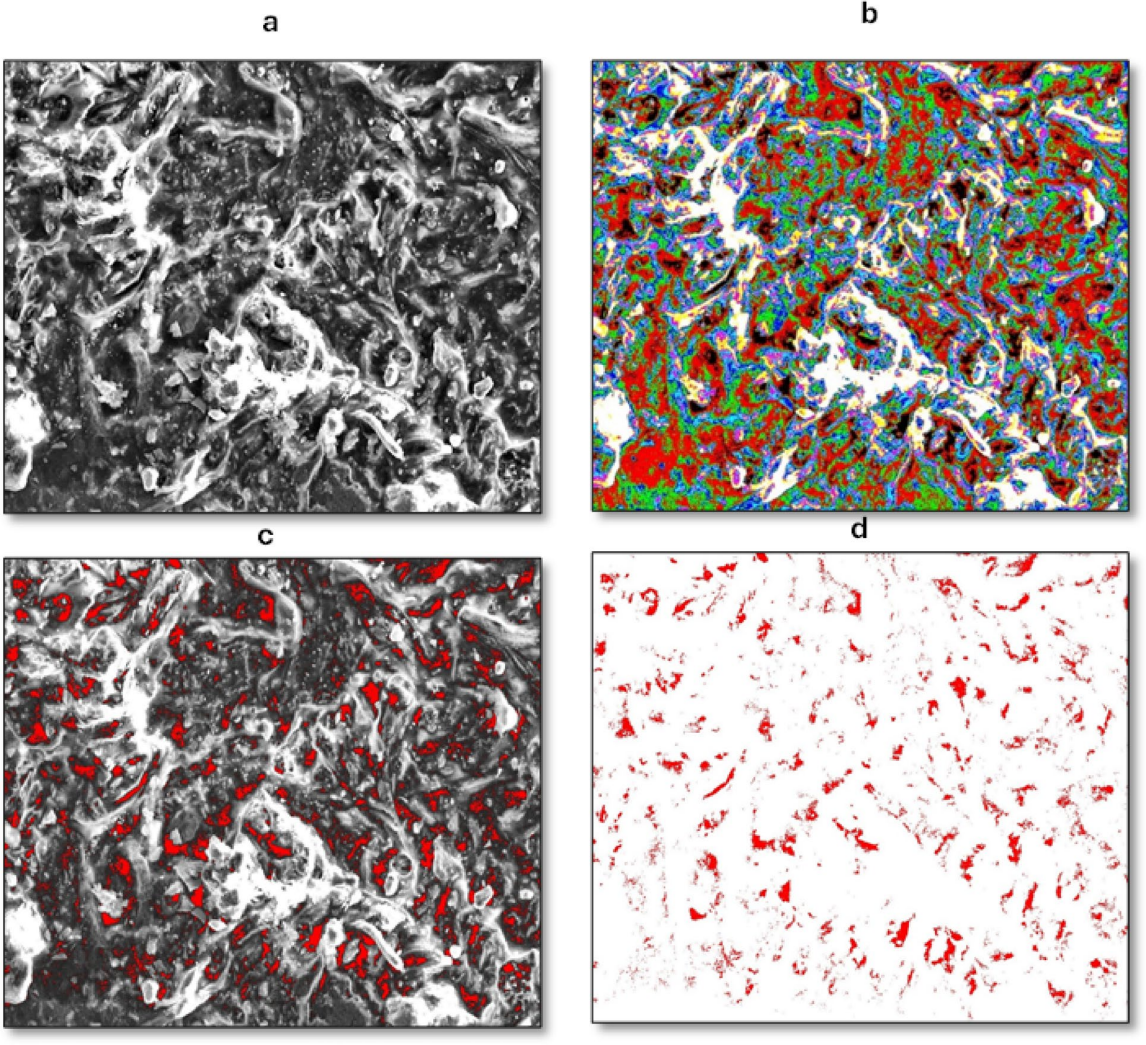
(**a**) P-pot. (**b**) BRGBCMYW incorporation. (**c**) Thresholding. (**d**) Void detection.

**Fig. 13:**
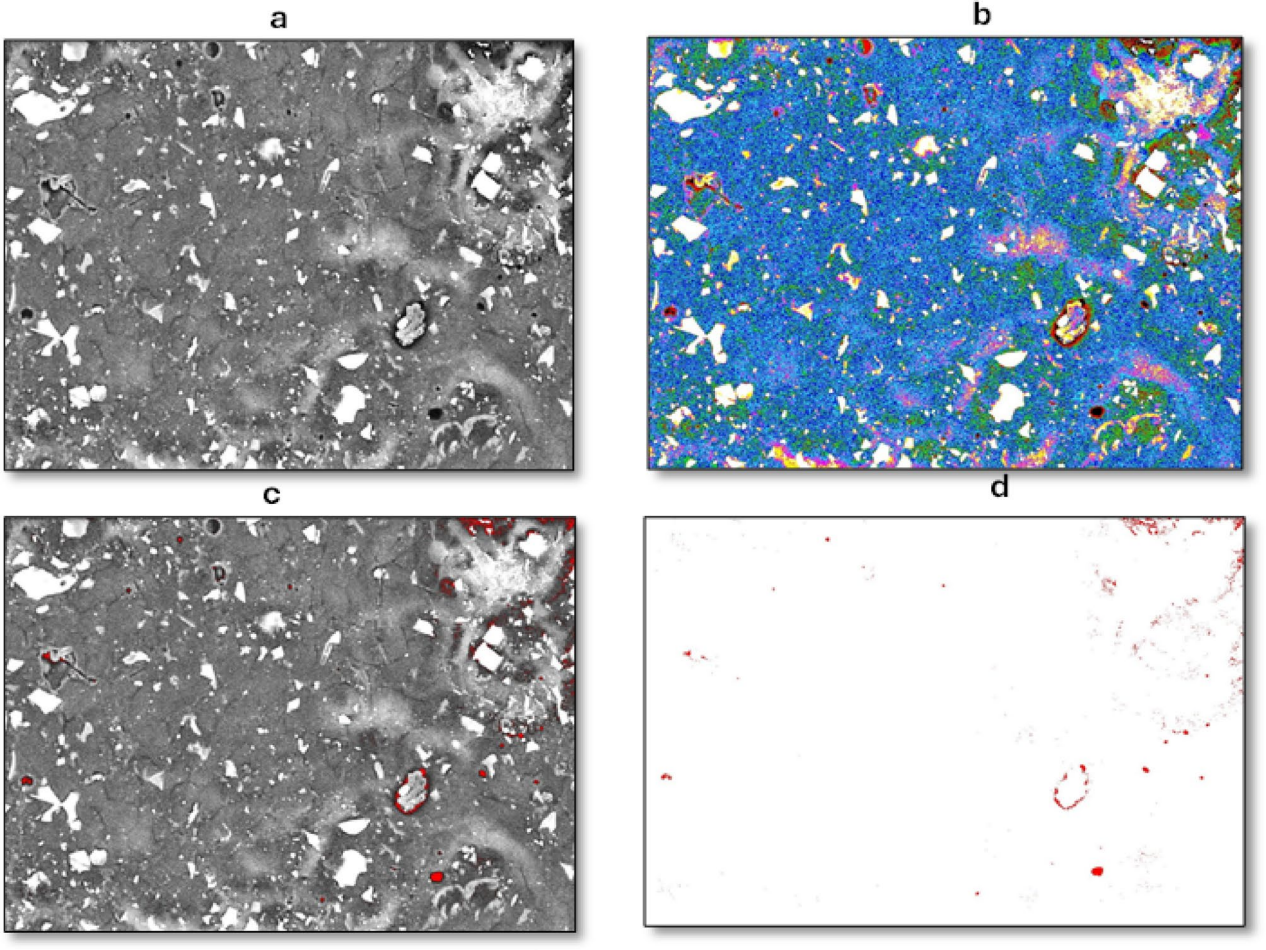
(**a**) PW-pot. (**b**) BRGBCMYW incorporation. (**c**) Thresholding. (**d**) Void detection.

**Fig. 14:**
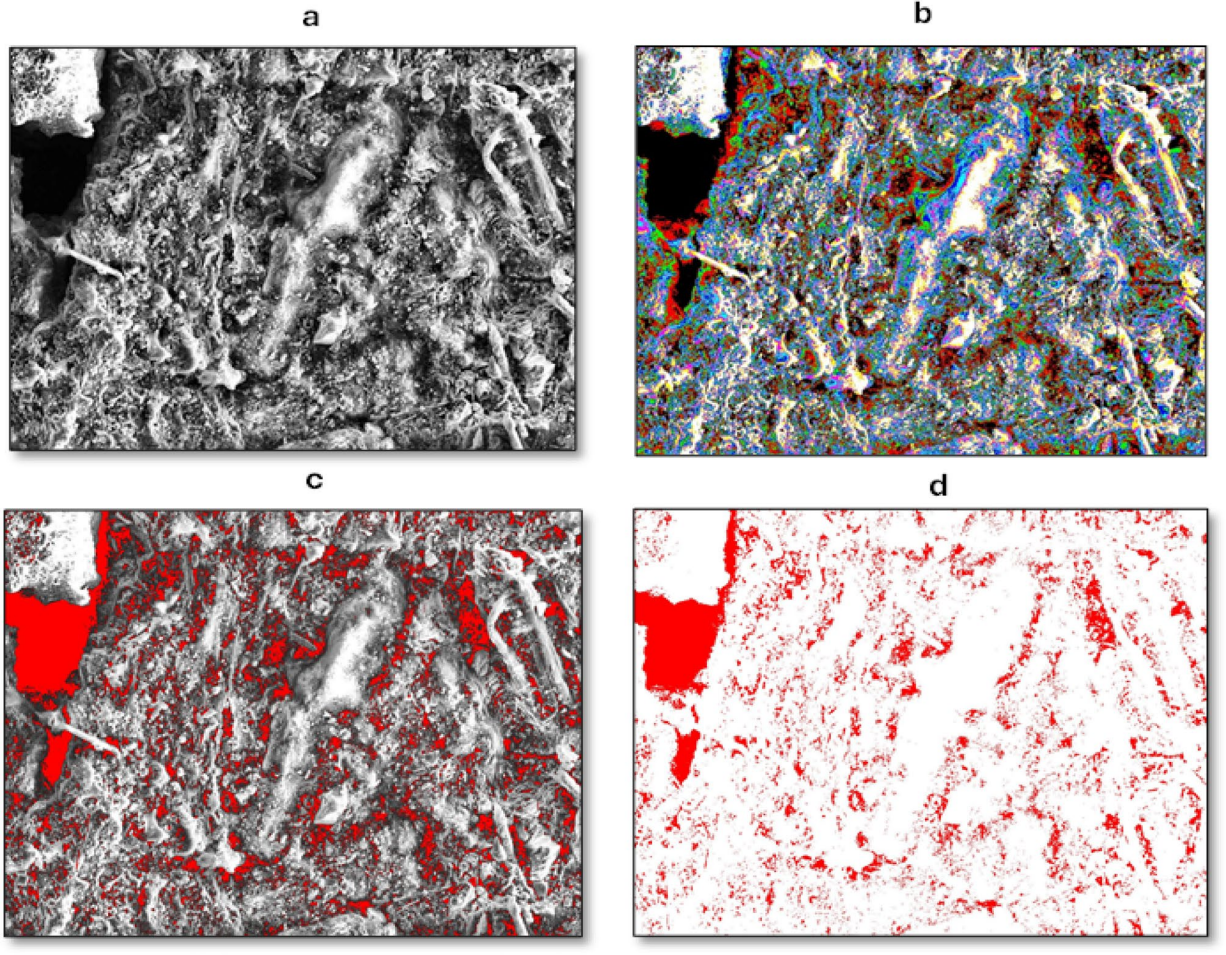
(**a**) L-pot. (**b**) BRGBCMYW incorporation. (**c**) Thresholding. (**d**) Void detection.

**Fig. 15:**
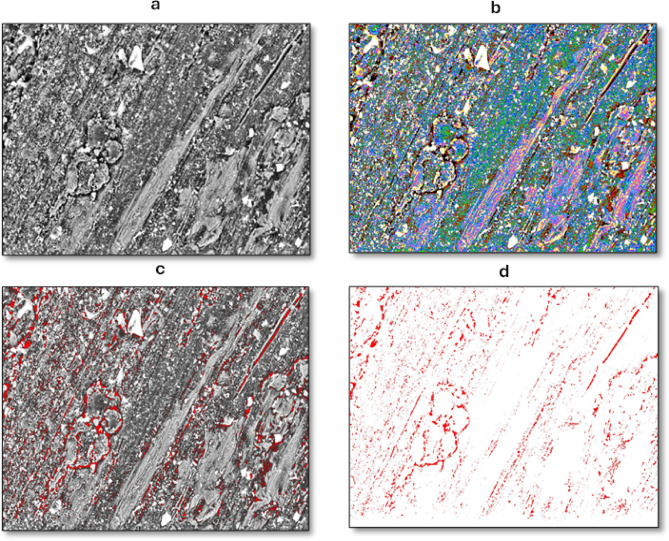
(**a**) LW-pot. (**b**) BRGBCMYW incorporation. (**c**) Thresholding. (**d**) Void detection.

Surface porosity was quantified using ImageJ through an intensity-based image analysis approach, as illustrated in Figs. 12, 13, 14, and 15. SEM micrographs were first converted to 8-bit grayscale and calibrated using the scale bar provided in each image.

A consistent threshold range was applied to distinguish low-intensity regions corresponding to voids (pores) from the solid matrix. Binary images were subsequently generated, in which pore regions were represented as black pixels and the solid phase as white pixels.

The percentage porosity was calculated as the ratio of the void (black) area to the total analyzed area. All measurements were performed in triplicate, and identical thresholding parameters were applied across all samples to ensure reproducibility and consistency.

It is challenging to define pore and void thresholds with the naked eye; therefore, to enhance visual discrimination of microstructural features, pseudo-color mapping using a look-up table (LUT) was applied. In this approach, grayscale intensity values are deterministically mapped to a color spectrum (e.g., BRGBCMYW), improving contrast and facilitating identification of low-density regions.

Based on intensity differences, low-intensity regions corresponding to voids were identified and subsequently segmented through thresholding. Binary images were then generated, in which pore regions were represented as black pixels and the solid matrix as white pixels.

However, pseudo-color mapping was used solely as a visualization tool and did not influence the quantitative analysis, which was based exclusively on intensity-driven thresholding and binary segmentation.

Quantification was based solely on intensity-driven thresholding followed by binary segmentation, consistent with established methodologies for area fraction analysis in composite materials^55–58^.

The segmentation approach was validated by maintaining consistent thresholding parameters across all samples and verifying that the extracted porosity trends agreed with the observed SEM morphology.

The surface porosity results in Table 7, revealed values of 5.97%, 0.43%, 12.51%, and 4.55% for P-, PW-, L-, and LW-pots, respectively. Statistical analysis indicated significant differences among the samples (*p* < 0.05).

**Table 7:** Surface porosity of cultivating pots samples estimated using ImageJ.

Type	Surface porosity (%)
P	5.979 ± 1.3^B^
PW	0.43 ± 0.31^C^
L	12.511 ± 1.7^A^
LW	4.555 ± 1^B^

Means that do not share a letter differ significantly using one-way ANOVA at *p* < 0.05 (Tukey’s HSD).

The PW-pot exhibited the lowest porosity, suggesting a more compact and homogeneous structure, while the L-pot showed the highest porosity, indicating increased structural heterogeneity.

Although the general trend is consistent with previous findings^8^, Some deviations were observed, particularly for PW and L samples. These discrepancies may be attributed to sample preparation artifacts, such as micro-fractures induced during cutting, or variations in surface roughness and topography affecting image segmentation.

#### Surface topography of cultivating pots

**Fig. 16:**
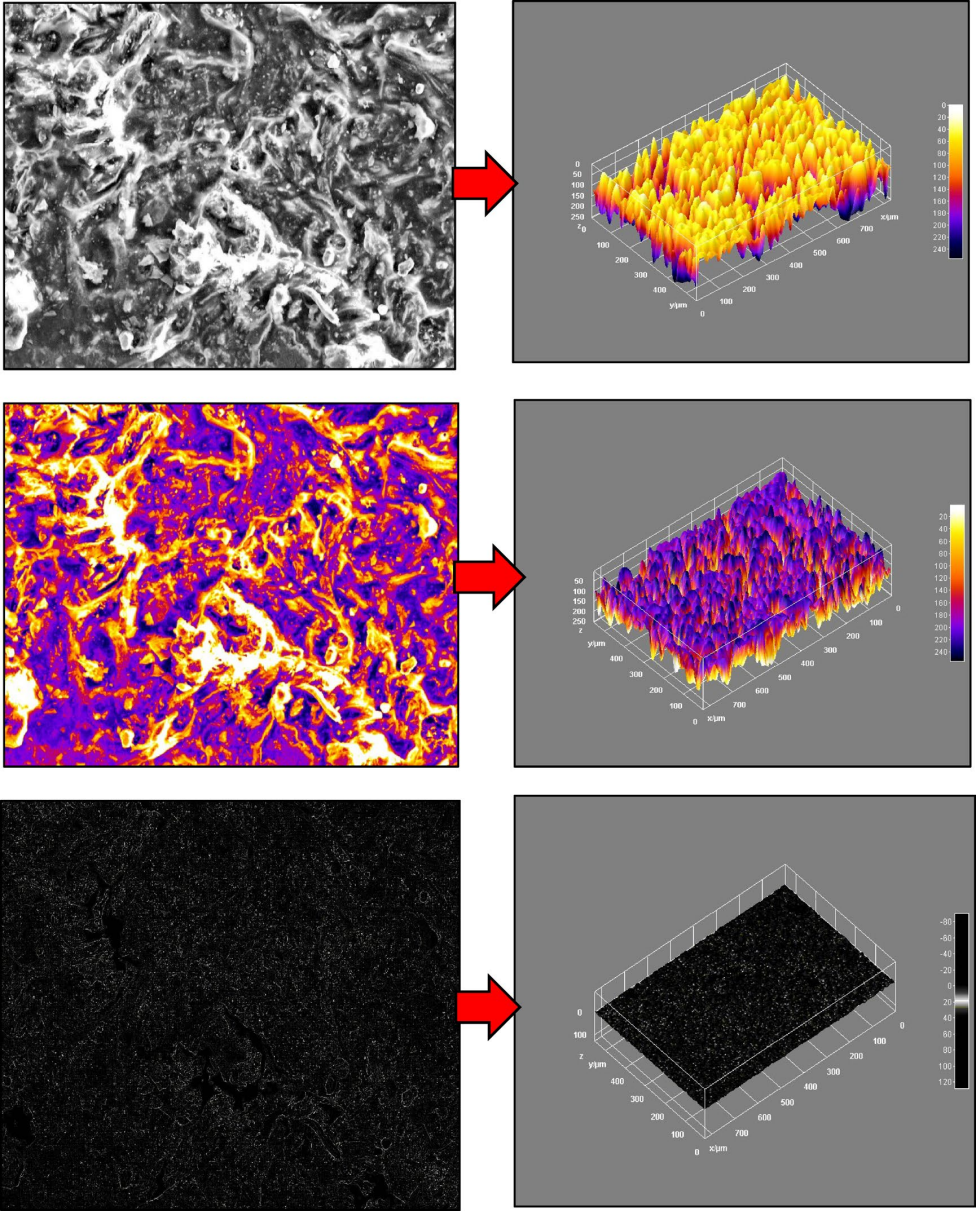
3D-topographical surface plot of P-pot, waviness, and roughness.

**Fig. 17:**
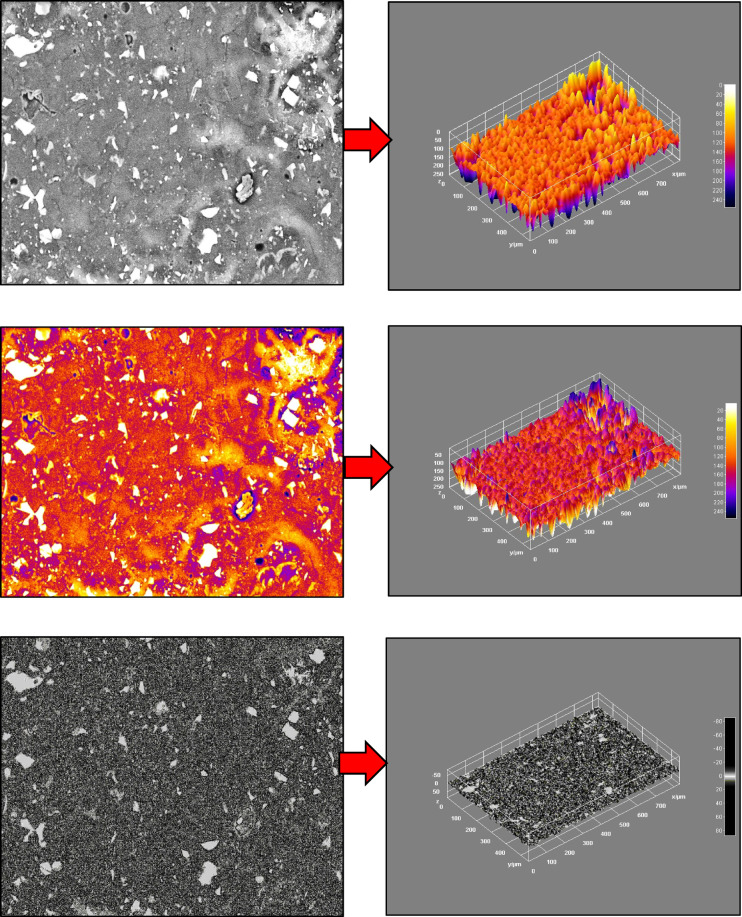
3D-topographical surface plot of PW-pot, waviness, and roughness.

**Fig. 18:**
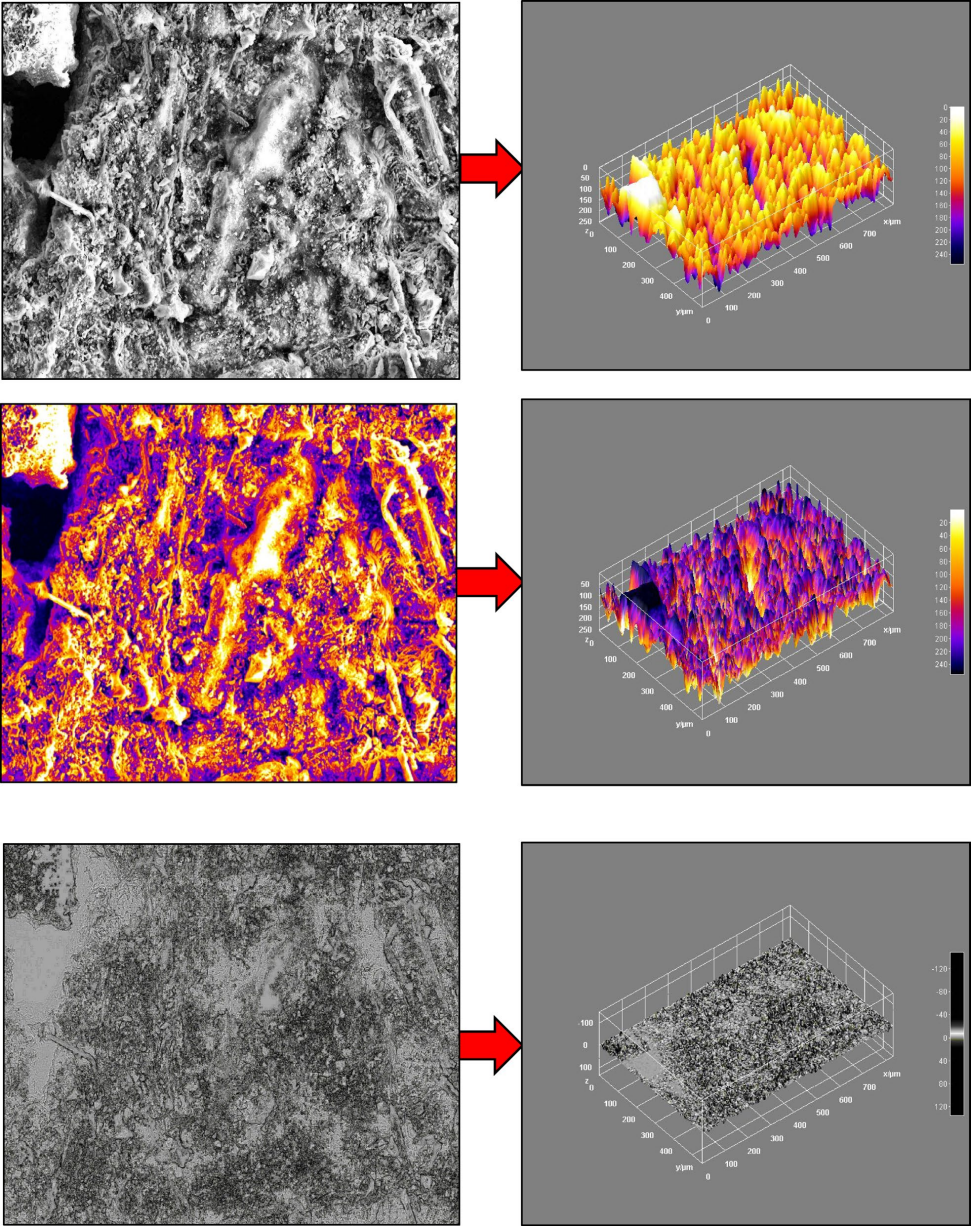
3D-topographical surface plot of L-pot, waviness, and roughness.

**Fig. 19:**
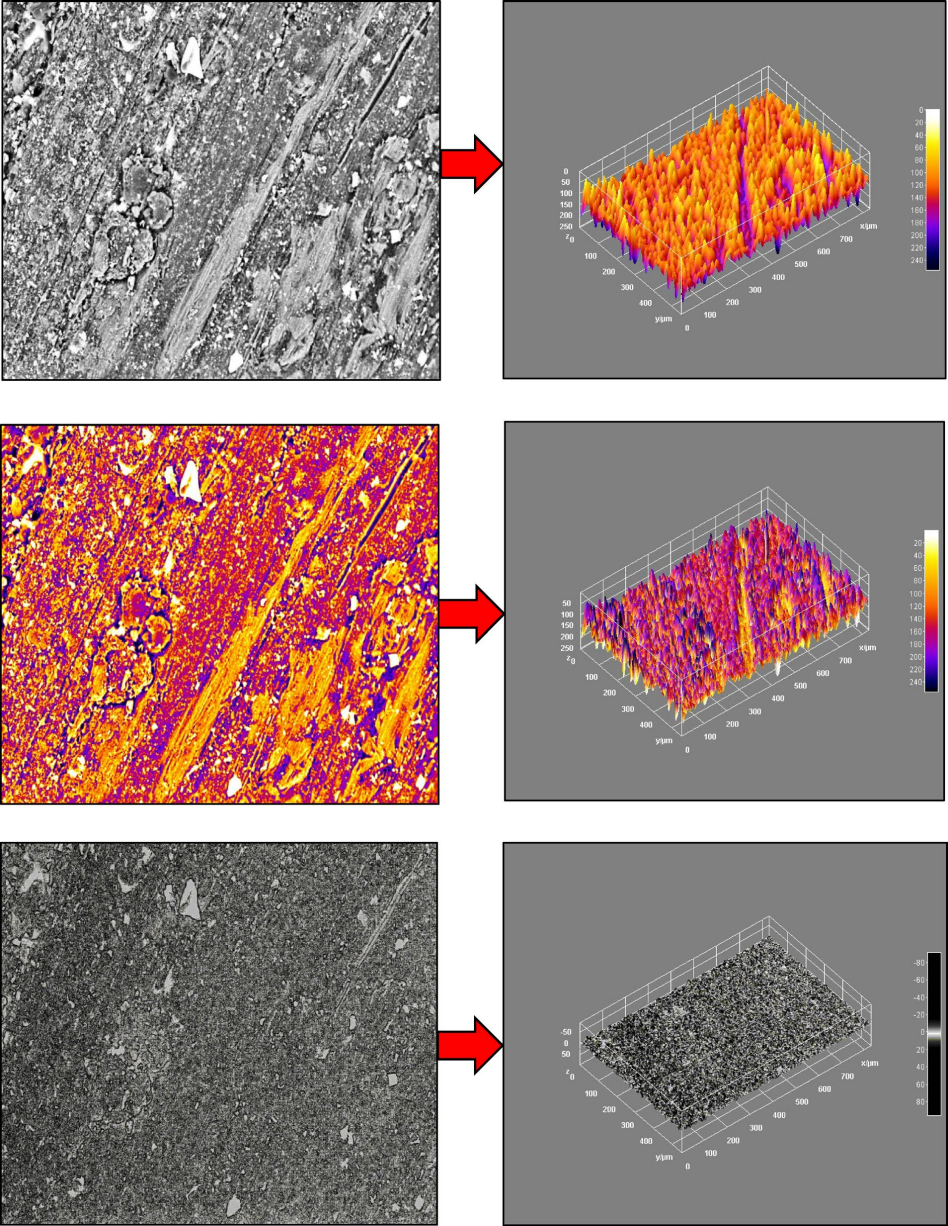
3D-topographical surface plot of LW-pot, waviness, and roughness.

ImageJ uses a Gaussian filter to decompose the surface into waviness and roughness. Therefore, a 3D-surface plot of samples, waviness, and roughness is shown in Figs. 16, 17, 18, and 19.

The roughness of cultivation pots was determined based on the data presented in Figs. 16, 17, 18, and 19 and summarized in Table 8. Palm wax exhibits rapid crystallization, forming molecular aggregates. Weak, nonpolar interactions between wax globules and composite molecules occur due to flocculation and coalescence of wax globules, leading to crack formation within the matrix, as depicted in Fig. 5. The observed roughness, attributed to the insolubility of wax, aligns with findings reported by^59^.

**Table 8:** Surface roughness of cultivating pots.

Label	Mean of roughness ± SD (μm)
P	104.678 ± 7.383^A^
PW	122.661 ± 20.598^A^
L	133.318 ± 13.742^A^
LW	124.87 ± 17.305^A^

Means that do not share a letter differ significantly using one-way ANOVA at *p* < 0.05 (Tukey’s HSD).

#### 2-D fast Fourier transformation (FFT)

**Fig. 20:**
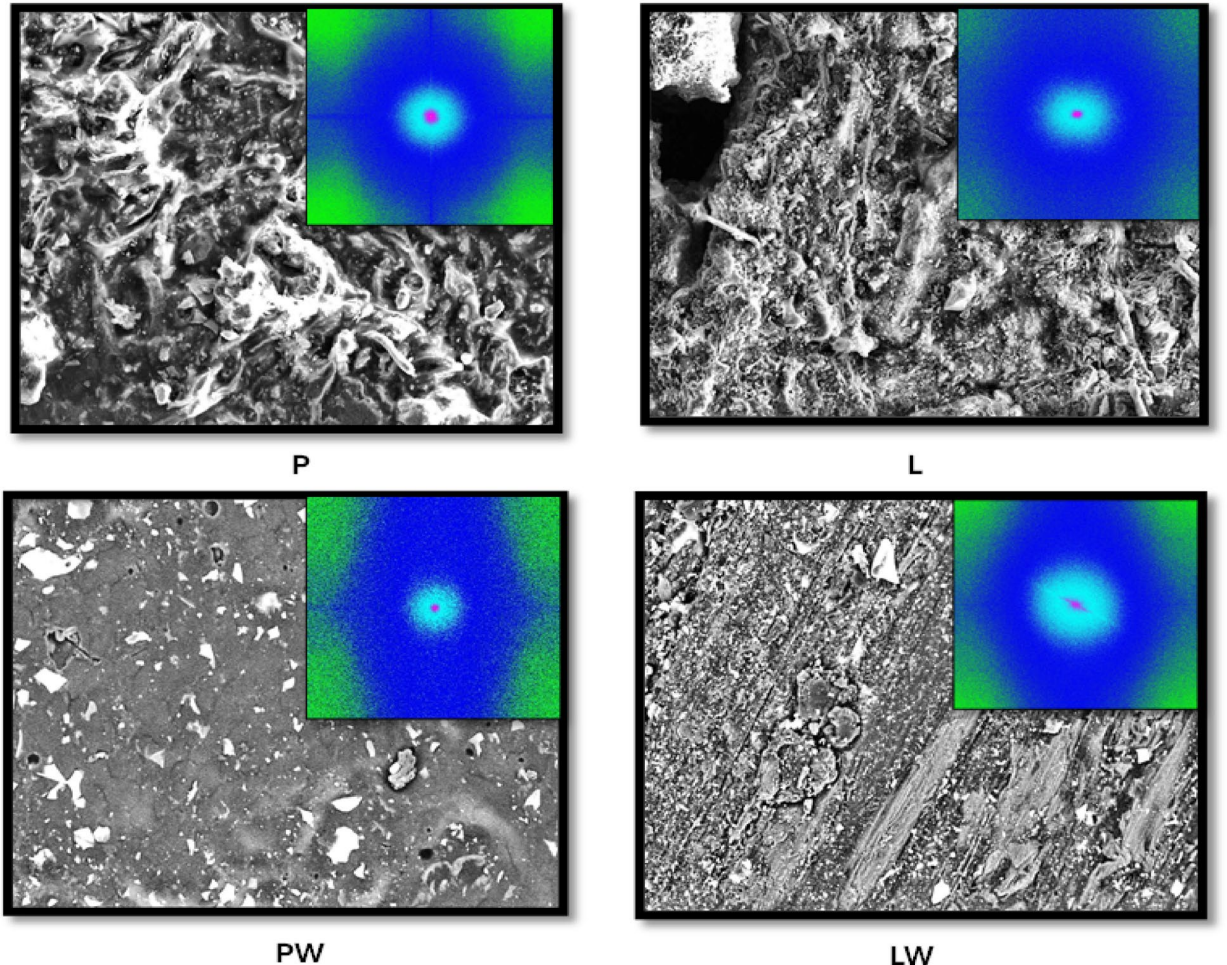
Fourier diffractograms of micrographs for cultivating pots.

The 2-D fast Fourier transformation (FFT) of the surfaces of the pots is depicted in Fig. 20. This filter provides a frequency domain representation by computing a convolution in the Fourier domain. It is considered a summation of harmonic functions with various amplitudes and phases of micrographs.

The mechanical performance, biodegradation behavior, and agronomic effects of the developed biocomposite cultivation pots have been investigated in our previous studies^8,9^. Those studies demonstrated adequate structural stability during cultivation, progressive biodegradation under soil conditions, and enhanced plant growth performance, including yield improvements of up to 13% in Anaheim chili pepper cultivation. Building upon these findings, the present study focused primarily on detailed structural and microstructural characterization to provide deeper insight into the mechanisms governing material performance and functionality.

## Conclusion

Biodegradable biocomposite cultivation pots reinforced with mercerized sugarcane bagasse and natural fillers were successfully developed and comprehensively characterized through chemical, structural, microstructural, and agronomic evaluations. Alkaline treatment using NaOH significantly modified the fiber structure through partial removal of lignin and hemicellulose, as confirmed by FTIR and XRD analyses, resulting in improved fiber–matrix interaction and enhanced microstructural characteristics.

Quantitative image analysis of SEM micrographs revealed notable differences among formulations, enabling assessment of homogeneity, porosity, grain intensity distribution, surface topography, and frequency-domain characteristics. The treated formulations exhibited improved structural uniformity and reduced surface porosity, indicating enhanced material integrity and compatibility between the reinforcing fibers and binder matrix.

In this study, pseudo-color mapping was applied as a contrast enhancement tool to facilitate improved identification of low-intensity regions corresponding to voids. While this approach enhanced visual discrimination, quantitative analysis was based on intensity-driven thresholding and binary segmentation, ensuring consistency with established image analysis methodologies. The integration of conventional materials characterization with advanced image-based analysis provided a systematic framework for evaluating biocomposite cultivation systems and identifying structure–property relationships.

The developed pots also demonstrated favorable physicochemical properties, including suitable pH and electrical conductivity conditions for plant cultivation. Greenhouse experiments using Anaheim chili pepper (Capsicum annuum) confirmed the agricultural potential of the developed materials, with the optimized formulation producing approximately 13% greater yield than the control treatment under the tested conditions. The biodegradable pots may gradually release organic constituents into the surrounding substrate, contributing to nutrient availability and improved rhizosphere conditions.

Beyond material performance, the utilization of sugarcane bagasse and other renewable resources contributes to the valorization of agricultural residues and supports the development of sustainable alternatives to conventional petroleum-based plastic pots. The proposed characterization methodology further provides a practical approach for designing and optimizing biodegradable cultivation containers tailored to specific agricultural applications.

Although the results demonstrated the potential of the developed biocomposite, future research should include field-scale validation, evaluation of additional crops, long-term biodegradation monitoring to validate their broader agricultural applicability, life-cycle assessment, and techno-economic analysis to further establish the environmental, agronomic, and commercial feasibility of the developed biocomposite cultivation pots.

Overall, the present study highlights the potential of structurally engineered biocomposite materials as sustainable alternatives for agricultural cultivation systems, while providing a systematic methodology for structural characterization and performance evaluation.

## Data Availability

The datasets used or analyzed during the current study are available from the corresponding author upon reasonable request.
